# Syphilis Exposure During Pregnancy and Childhood Hospital Admissions in Brazil

**DOI:** 10.1001/jamanetworkopen.2025.7471

**Published:** 2025-04-30

**Authors:** Enny S. Paixão, Orlagh Carroll, Laura C. Rodrigues, Guilherme Lopes de Oliveira, Andrey Moreira Cardoso, Rita de Cássia Ribeiro-Silva, Mauricio L. Barreto, Maria Yury Ichihara

**Affiliations:** 1 Faculty of Epidemiology and Population Health, London School of Hygiene and Tropical Medicine, London, United Kingdom; 2 Centre for Data and Knowledge Integration for Health (CIDACS), Fundação Oswaldo Cruz, Salvador, Brazil; 3 Centro Federal de Educação Tecnológica de Minas Gerais, Belo Horizonte, Minas Gerais, Brazil; 4 Escola Nacional de Saúde Pública, Rio de Janeiro, Brazil

## Abstract

**Importance:**

While the short-term impacts of congenital syphilis are well documented, the long-term outcomes and effects on children exposed to syphilis during pregnancy but without congenital infection detected at birth remain unclear.

**Objective:**

To compare the rates of all-cause hospitalization in children younger than 5 years between those exposed to syphilis during pregnancy (with and without congenital syphilis) and those unexposed to syphilis during pregnancy.

**Design, Setting, and Participants:**

Population-based study using linked data from the Center of Data and Knowledge Integration for Health Birth Cohort, including singleton live births between January 1, 2011, and December 31, 2015. Children were followed up until age 5 years, death, or December 31, 2018, whichever occurred first. Data were made available in 2020 and analyzed between March and September 2024.

**Exposures:**

In this study, syphilis during pregnancy was categorized into 3 groups: (1) maternal syphilis, (2) congenital syphilis, and (3) no exposure, for those without syphilis records.

**Main Outcomes and Measures:**

Primary outcomes were: (1) general and age-stratified first hospital admission rates, (2) length of stay for the first hospital admission, and (3) *International Classification of Diseases and Related Health Problems, Tenth Revision *diagnoses of the first hospitalization. Secondary outcomes included: (1) recurrent hospital admissions and (2) mortality.

**Results:**

The study included 8 286 867 singleton births, with 30 039 exposed to maternal syphilis (15 482 [51.5%] male, 7642 [25.4%] Asian, 2442 [8.1%] Black, and 16 930 [56.4%] Pardo [denotes individuals who are White and Indigenous, White and Black, Black and Indigenous, or Black and another race]) and 36 443 with congenital syphilis (18 286 [50.2%] male, 6977 [19.1%] Asian, 3769 [10.3%] Black, 16 930 [56.4%] Pardo). Exposed births were more prevalent in Black, single, and less educated women. Compared with children with no exposure, those with congenital syphilis had an increased risk of first hospitalization (hazard ratio [HR], 6.19; 95% CI, 6.11-6.28), as did those exposed to maternal syphilis (HR, 1.90; 95% CI, 1.86-1.94). The highest risk of first hospitalization was observed in the first month of life, among those with congenital syphilis (HR, 11.53; 95% CI, 11.36-11.70). Although the risk decreased with age, children exposed to syphilis continued to have higher hospitalization rates than the nonexposed group until the age of 36 months. Live-born children exposed to syphilis during pregnancy also had more and longer hospital admissions. Those with congenital syphilis had a lower prevalence of respiratory and digestive diagnoses than those with maternal syphilis and the nonexposed group.

**Conclusions and Relevance:**

In this cohort study, we observed that those children exposed to syphilis during pregnancy, even without congenital syphilis detected at birth, had a higher risk of hospital admission and were hospitalized for longer periods. These findings underscore the need for close monitoring of exposed children and emphasize the importance of preventing syphilis in women of childbearing age.

## Key Points

**Question:**

Does syphilis during pregnancy (with and without congenital syphilis compared with no syphilis exposure) increase the risk of all-cause hospital admission in children under five years?

**Findings:**

This cohort study of 8 286 867 singleton births found that children with congenital syphilis faced a 6-fold increased hazard of first hospitalization, while those exposed to maternal syphilis had nearly a 2-fold increase compared with nonexposed children. Overall, children exposed to syphilis experienced more and longer hospital admissions than their counterparts.

**Meaning:**

These results suggest the need for close monitoring of exposed children and stress the importance of preventing syphilis in women of childbearing age.

## Teaser

This cohort study compares rates of all-cause hospitalization in children exposed to syphilis during pregnancy vs those unexposed in a population-based Brazilian cohort.

## Introduction

Syphilis, a bacterial infection caused by *Treponema pallidum*, once primarily endemic to low- and middle-income countries, has recently emerged as a significant global public health challenge. Its incidence has increased in high-income countries^1^ with rates in women of childbearing age rising by more than 200%^2^ in recent years. Syphilis can be transmitted sexually or vertically from mother to child during pregnancy, affecting the developing fetus.^3^ It is estimated that globally, maternal syphilis has caused 350 000 adverse birth outcomes every year, including stillbirth, preterm birth, low birth weight, neonatal deaths, and congenital infection.^4^

Early detection and timely treatment during antenatal care are effective interventions that can substantially reduce adverse pregnancy outcomes.^3^ A meta-analysis estimated that long-acting penicillin regimens in pregnancy reduce the relative risk of congenital syphilis by 97%, stillbirths by 82%, preterm delivery by 64%, and neonatal death by 80%.^5,6,7^ Despite the success of treatment, concerns remain regarding the potential adverse consequences of syphilis exposure during pregnancy.

Studies have shown the impact of congenital syphilis on child health, including ocular abnormalities, hearing loss, bone damage, skin lesions, higher mortality rates, and longer hospital stays.^8,9,10^ Few studies have investigated the health outcomes of children exposed to syphilis during pregnancy but without congenital infection detected at birth. Additionally, many studies have had short follow-up (mainly during the neonatal period), high attrition rates, and small sample sizes.^8^ Therefore, uncertainty remains regarding the health outcomes of children exposed to syphilis during pregnancy. Understanding the health risks for children exposed during pregnancy is an important step in optimizing child health in areas with high syphilis burden.

In this study, we aim to compare the rates of all-cause hospitalization in children under 5 years between those exposed to syphilis during pregnancy (with and without congenital syphilis) and those unexposed to syphilis during pregnancy. Additionally, we will examine recurrent hospital admission and death.

## Methods

### Study Population and Data Source

This study received approval from the research ethics committee of the Federal University of Bahia. While informed consent was waived, authorization was obtained from the legal guardian of the data, the Brazilian Ministry of Health, to conduct this study. The study is reported in accordance with the Reporting of Studies Conducted Using Observational Routinely-Collected Data (RECORD) guideline.^11^

This study included singleton live births from the Center of Data and Knowledge Integration for Health (CIDACS) Birth Cohort between January 1, 2011, and December 31, 2015. These children were followed up from birth until age 5 years, death, or December 31, 2018, whichever occurred first. The CIDACS Birth Cohort includes intersecting records from the Unified Register for Social Programs (CadÚnico) and the Live Birth Information System (SINASC) since 2001 in Brazil. As the CadÚnico represents individuals who have applied for social assistance nationwide, this cohort reflects the poorest segment of the Brazilian population. Live-born children from this cohort are more likely to have younger, unmarried mothers with fewer years of education compared with the general population of live births in Brazil.^12^ We have included data from 2011 in this analysis due to the availability of certain variables from SINASC that were included in the updated 2011 version.

The CIDACS Birth Cohort data were linked to the Information System for Notifiable Diseases (SINAN-Syphilis), the Hospital Information System (SIH) and the Mortality Information System (SIM). Detailed information about data sources appear in eTable 1 in Supplement 1.^12,13,14^

Singleton births were excluded from the study population due to mismatched birth dates across datasets, date of death before date of birth or hospital admission, or if hospital admission date was before date of birth. Additionally, those with a hospital stay of 0, more than 360 days, or those missing gestational age at birth were excluded.

### Linkage Process

CIDACS Birth Cohort records were linked separately to SINAN maternal syphilis, SINAN congenital syphilis, SIH, and SIM records using variables such as maternal name, date of birth, age, and place of residency as matching criteria. This linkage was performed using CIDACS-Record Linkage, a novel tool developed at CIDACS. CIDACS-Record Linkage employs a combination of indexing and searching algorithms to identify records from the Birth Cohort that closely match each record in the remaining datasets. It proceeds with pairwise comparisons of candidate linking records, ordering them based on scores, and retaining only the pair with the highest score as a potential link.^15^ The accuracy of each linkage was assessed through manual verification of a random sample of records, evaluating sensitivity and specificity indexes via receiver operating characteristic curves. For linkage between SINASC and SIM, a sample of 2000 pairs stratified into 3 categories of linkage score (high: >0.95, intermediate: 0.90-0.95, and low: <0.90) was obtained and manually reviewed to evaluate linkage quality. We obtained a mean sensitivity and specificity greater than 93% in this validation process. For linkage between SINASC and SIH, the sensitivity and specificity were higher than 97% in all years. For linkage between SINASC and SINAN-Syphilis, the sensitivity and specificity were above 91%. All linkage procedures were conducted at CIDACS under strict data protection measures and in compliance with Brazil’s ethical and legal regulations.^16^

### Exposure and Outcomes Definition

In Brazil, maternal syphilis is defined based on criteria that combine information from laboratory tests, symptoms, and treatment. Women meeting 1 of the following combinations during prenatal care, childbirth, or the puerperium period are reported as having maternal syphilis: asymptomatic women with at least 1 positive laboratory test for syphilis without prior treatment; symptomatic women with at least 1 positive laboratory test for syphilis, regardless of prior treatment; or women with positive results on both nontreponemal and treponemal tests, regardless of treatment status or symptoms.

According to the Brazilian Ministry of Health, individuals meeting 1 or more of the following criteria should be reported as a congenital syphilis case: live births from mothers with untreated or inadequately treated syphilis; children with microbiological evidence of *Treponema pallidum* in nasal discharge, skin lesion, child biopsy, or autopsy; or children less than 13 years with at least 1 of the following: clinical, cerebrospinal fluid, or radiological manifestations of congenital syphilis and a positive nontreponemal test; infants (younger than 1 year) with nontreponemal test titers greater than those of the mother in at least 2 dilutions; children with increasing nontreponemal test titers in at least 2 dilutions; nontreponemal test titers remaining positive in a child older than 6 months who was adequately treated in the neonatal period; or a positive treponemal test in a child aged 18 months without a previous diagnosis of congenital syphilis.

The exposure, syphilis during pregnancy, was classified into 3 categories. The first was maternal syphilis: live births exposed to maternal syphilis but without congenital syphilis reported at birth, defined as any record from SINAN gestational syphilis linked with a record of singleton live births from the CIDACS Birth Cohort, where the maternal syphilis notification from SINAN occurred between the conception date of the live birth and the end of the puerperium period. The live birth record must not be linked to a congenital syphilis record from SINAN. The second was congenital syphilis, or live births with congenital syphilis; defined as records from SINAN congenital syphilis linked with a record of singleton live births from the CIDACS Birth Cohort, where the syphilis notification from SINAN occurred less than 2 months before birth and no more than 3 years after birth. The third was those without a link to SINAN syphilis records were classified as nonexposed to syphilis during pregnancy. We compared (1) congenital syphilis vs nonexposed, (2) maternal syphilis vs nonexposed, (3) any syphilis exposure vs nonexposed.

Our primary outcomes were: (1) general and age-stratified first hospital admission; (2) length of stay in the first hospital admission; and (3) first hospital admission causes classified by *International Classification of Diseases and Related Health Problems, Tenth Revision (ICD-10)*.^17^ Secondary outcomes included: (1) recurrent hospital admission as a time-to-event outcome and (2) mortality. All hospital entries from birth up to 5 years were included. To account for failed discharge, readmissions occurring within 6 days were considered to be a continuation of the previous hospital episode. We identified live-born children who were admitted to a hospital by linking SIH with the CIDACS Birth Cohort.

### Covariates

Following previous research, we include maternal region of residence (North, Northeast, South, Southeast, or Central-West), year of birth, age of mother, relationship status of mother (in a relationship or single), mother’s education (none, 1-3 years, 4-7 years, 8-11 years, or ≥12 years) and race and ethnicity (Asian, Black, Indigenous, Pardo [denotes individuals who are White and Indigenous; White and Black; Black and Indigenous; or Black and another race], or White) as potential confounders.^9,18,19^ Race and ethnicity were patient-reported and were assessed because they are important social determinants of health in Brazil, and the impact of racial disparities on syphilis outcomes has been observed before.^20^

### Statistical Analysis

Baseline characteristics are presented by syphilis status at birth (congenital, maternal, or nonexposed). All *ICD-10* codes from the first hospital admission are summarized using a Sankey plot.

The analysis for each outcome involved running a regression model adjusted for the covariates previously mentioned and 2 binary indicators for having either congenital or maternal syphilis vs the nonexposed group. To estimate any syphilis exposure, the mean of the binary indicators’ parameters was taken on the log-scale and exponentiated.

For time to first hospitalization and death, we summarize the median (IQR) of the follow-up time in months. We used Kaplan-Meier and a multivariable Cox proportional hazards model adjusted for syphilis status and the confounders defined above. Missing values in age, marital status and education of the mother at time of birth were minimal and deemed unlikely to be associated with the outcomes being evaluated; a complete-case analysis was used.^21^ The proportional hazards assumption was checked using Schoenfeld residuals. We reported hazard ratios (HR) overall and by age (1, 12, 24, 36, 48 and 60 months).

For all-cause hospitalizations, we used the Andersen and Gill model^22^ to account for repeated admissions. This was adjusted for syphilis status, covariates, and the number of previous hospital admissions. Hospitalizations beyond the sixth occurrence were not considered to ensure a sufficient sample size.^23^ For patients with a hospital admission, the first hospital length of stay was analyzed using Gamma regression adjusting for syphilis and covariates. The mean difference in days and 95% CIs were reported.

Supplementary analyses for all regression analyses included (1) restricting to children with birthweight 2500 to 6000 g and (2) excluding those born before 37 weeks (preterm). Analyses were conducted using R 4.3.3 (R Project for Statistical Computing),^24^ and the survival,^25^ ggsankey,^26^ tableone^27^ and data.table packages.^28^ Data were analyzed between March and September 2024.

## Results

### Baseline Characteristics

**Figure 1.**
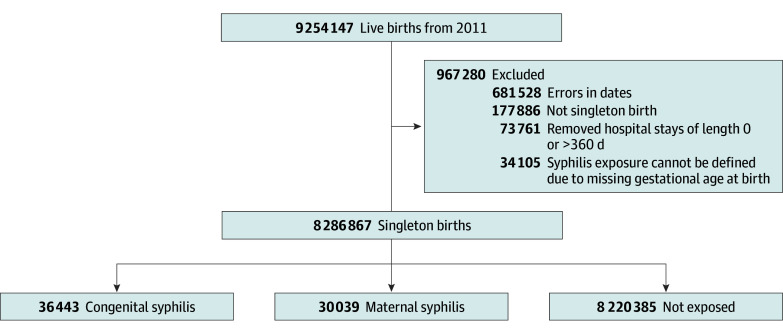
Flow Diagram Illustrating Inclusion of Live Births in the Study Cohort

We included 8 286 867 singleton births in our study (Figure 1). Among these, 66 482 were exposed to syphilis during pregnancy. Specifically, 30 039 (0.4%) had maternal syphilis (congenital syphilis not detected at birth), and 36 443 (0.4%) had congenital syphilis. The characteristics of the study population are detailed in the Table. Live births exposed to syphilis during pregnancy were more likely to be from Black, single, and less educated women compared with those nonexposed. A total of 5730 of 36 443 (15.7%) and 2871 of 30 039 (9.6%) children were born preterm, and 6151 of 36 438 (16.9%) and 2772 of 30 015 (9.2%) had low birth weight for congenital and maternal syphilis, respectively. In comparison, the nonexposed group had 774 909 preterm births of 8 220 385 total births (9.5%) and 554 557 with low birth weight of 8 214 137 total births (6.7%).

**Table.** Baseline Characteristics of Singleton Live Births From January 1, 2011, to December 31, 2015

Variable	Participants, No. (%)^a^		
	Congenital (n = 36 443)	Maternal (n = 30 039)	Not exposed (n = 8 220 385)
Mother’s region of residence			
Central-West	1943 (5.3)	3171 (10.6)	623 661 (7.6)
Northeast	11 629 (31.9)	6540 (21.8)	2 938 522 (35.7)
North	3119 (8.6)	2988 (9.9)	1 032 482 (12.6)
Southeast	15 323 (42.0)	13 246 (44.1)	2 726 831 (33.2)
South	4429 (12.2)	4094 (13.6)	298 889 (10.9)
Mother’s age, y			
Mean (SD)	24.4 (6.4)	25.6 (6.8)	24.7 (6.4)
Median (IQR)	23.0 (19.0-29.0)	25.0 (20.0-30.0)	24.0 (20.0-29.0)
Age group			
<20	9239 (25.4)	6498 (21.6)	1 965 690 (23.9)
20-34	24 135 (66.2)	19 932 (66.4)	5 560 005 (67.6)
≥35	3069 (8.4)	3609 (12.0)	694 657 (8.5)
Missing	0	0	33
Mother’s education, y			
None	499 (1.4)	347 (1.2)	75 952 (0.9)
1-3	2599 (7.1)	1819 (6.1)	416 506 (5.1)
4-7	14 690 (40.3)	9845 (32.8)	2 237 775 (27.2)
8-11	17 414 (47.8)	16 467 (54.8)	4 845 730 (58.9)
≥12	708 (1.9)	1061 (3.5)	474 165 (5.8)
Missing	533 (1.5)	500 (1.7)	170 257 (2.1)
Sex			
Female	18 148 (49.8)	14 553 (48.4)	4 042 151 (49.2)
Male	18 286 (50.2)	15 482 (51.5)	4 176 962 (50.8)
Missing	9 (<0.1)	4 (<0.1)	1272 (<0.1)
Mother’s ethnicity			
Asian	6977 (19.1)	7642 (25.4)	1 945 106 (23.7)
Black	3769 (10.3)	2442 (8.1)	454 259 (5.5)
Indigenous	88 (0.2)	83 (0.3)	20 888 (0.3)
White	133 (0.4)	198 (0.7)	70 020 (0.9)
Pardo^b^	22 452 (61.6)	16 930 (56.4)	4 825 045 (58.7)
Missing	3024 (8.3)	2744 (9.1)	905 067 (11.0)
Mother’s relationship status			
In relationship	11 097 (30.5)	11 292 (37.6)	4 143 468 (50.4)
Single^c^	25 006 (68.6)	18 401 (61.3)	3 965 019 (48.2)
Missing	340 (0.9)	346 (1.2)	111 898 (1.4)
Delivery type			
Cesarean	13 218 (36.3)	13 417 (44.7)	3 885 204 (47.3)
Vaginal	23 183 (63.6)	16 590 (55.2)	4 324 517 (52.6)
Missing	42 (0.1)	32 (0.1)	10 664 (0.1)
Birth weight, g			
Mean (SD)	3016.4 (627.2)	3171.7 (570.3)	3216.9 (528.6)
Median (IQR)	3075.0 (2690.0-3420.0)	3200.0 (2875.0-3525.0)	3230.0 (2930.0-3540.0)
Weight group			
<1500	876 (2.4)	397 (1.3)	75 533 (0.9)
1500-2499	5275 (14.5)	2375 (7.9)	479 024 (5.8)
2500-5999	30 286 (83.1)	27 241 (90.7)	7 659 336 (93.2)
≥6000	0	0	244 (<0.1)
Missing	5 (<0.1)	24 (0.1)	6248 (0.1)
Gestational age at birth, wk			
Mean (SD)	38.2 (2.7)	38.7 (2.3)	38.7 (2.2)
Median (IQR)	39.0 (37.0-40.0))	39.0 (38.0-40.0)	39.0 (38.0-40.0)
Age group			
18-31	1018 (2.8)	410 (1.4)	94 812 (1.2)
32-36	4712 (12.9)	2461 (8.2)	680 097 (8.3)
37-44	27 639 (76.0)	23 261 (77.4)	6 524 498 (79.4)
No. of prenatal appointments			
Mean (SD)	6.0 (3.2)	7.6 (3.1)	7.1 (2.8)
Median (IQR)	6.0 (4.0-8.0)	8.0 (6.0-10.0)	7.0 (5.0-9.0)
Appointments by group			
0	2917 (8.0)	1028 (3.4)	221 635 (2.7)
1-3	6052 (16.6)	2205 (7.3)	684 197 (8.3)
4-6	11 888 (32.6)	7972 (26.5)	2 483 416 (30.2)
≥7	14 609 (40.1)	18 512 (61.6)	4 764 564 (58.0)
Missing	977 (2.7)	322 (1.1)	66 573 (0.8)
Year of birth			
2011	5047 (13.8)	3972 (13.2)	1 561 112 (19.0)
2012	5991 (16.4)	4637 (15.4)	1 586 364 (19.3)
2013	7191 (19.7)	5562 (18.5)	1 678 786 (20.4)
2014	8496 (23.3)	7203 (24.0)	1 758 553 (21.4)
2015	9718 (26.7)	8665 (28.8)	1 635 570 (19.9)
Syphilis classification^d^			
Primary	501 (1.4)	1016 (3.4)	0
Secondary	100 (0.3)	191 (0.6)	0
Tertiary	164 (0.5)	285 (1.0)	0
Latent	302 (0.8)	526 (1.8)	0
Missing	3 541 935 (97.2)	28 054 (93.4)	0

^a^ Counts less than 5 have been suppressed.

^b^ Pardo denotes individuals who are White and Indigenous, White and Black, Black and Indigenous, or Black and another race.

^c^ *Single* refers to those who are not in a relationship, separated, divorced or widowed.

^d^ A total of 57 congenital and 43 maternal syphilis cases had 1 to 3 *Treponema pallidum* test results.

During the study period, 23 733 of 36 443 live births with congenital syphilis (65.1%) and 9388 of 30 039 with maternal syphilis (31.3%) had at least 1 hospital admission, compared with 1 568 567 of 8 220 385 (19.0%) in the nonexposed group. Additionally, live births exposed to syphilis during pregnancy experienced longer hospital stays, with a median (IQR) duration of 8 (0-11) days for those with congenital syphilis, 0 (0-3) days for maternal syphilis (without congenital syphilis detected at birth), and 0 (0-0) days for the nonexposed group. Mortality rates were higher among those with congenital syphilis. Demographic information by number of hospital admissions and by survival status are available in eTables 2 and 3 in Supplement 1.

### First Hospitalization

**Figure 2.**
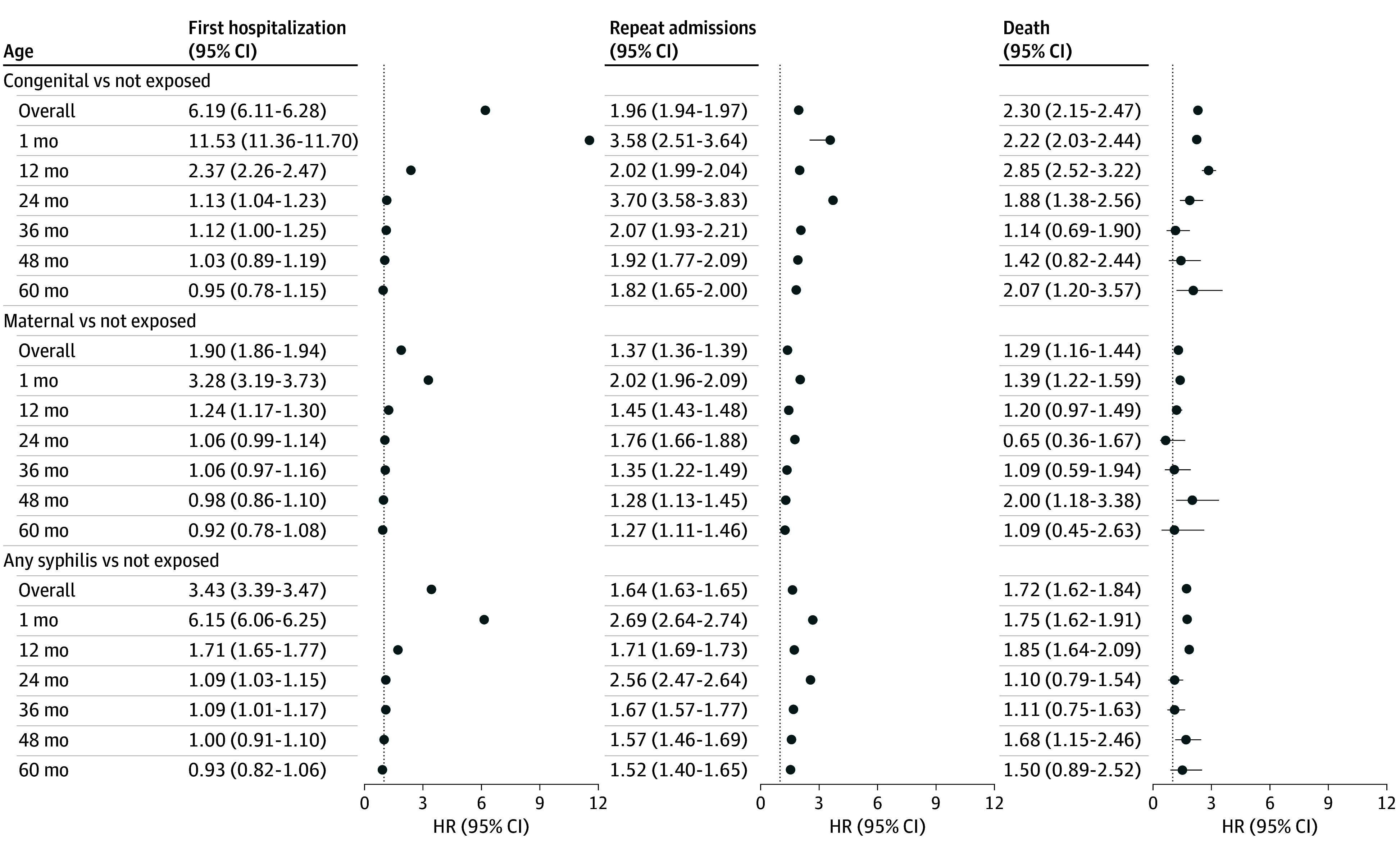
Forest Plot Showing Adjusted Hazard Ratios (HRs) Overall and Stratified by Age

The median (IQR) time to first hospitalization was 0.1 (0.0-48.0) months for congenital syphilis, 47.0 (10.5-60.0) months for maternal syphilis, and 57.5 (41.2-60.0) months for those nonexposed (See eTables 4 and 5 and eFigure 1 in Supplement 1). Over 5 years, those with congenital syphilis had a higher risk of being hospitalized compared with those nonexposed (HR, 6.19; 95% CI, 6.11-6.28). Those with maternal syphilis had an increased risk compared with the nonexposed group (HR, 1. 90; 95% CI, 1.86-1.94) (Figure 2; eTable 6 in Supplement 1).

The increased hazard of hospitalization was particularly high in the first month of life: children with congenital syphilis had 11 times the hazard of hospitalization of the nonexposed group (HR, 11.53; 95% CI, 11.36-11.70). Those with maternal syphilis had over 3 times the hazard of the nonexposed group (HR, 3.28; 95% CI, 3.19-3.73). An increased hazard was present for first hospitalization for the first 36 months of life for any syphilis exposure during pregnancy (congenital or maternal) compared with the nonexposed group, but this association decreased over time (Figure 2; eTable 7 in Supplement 1).

**Figure 3.**
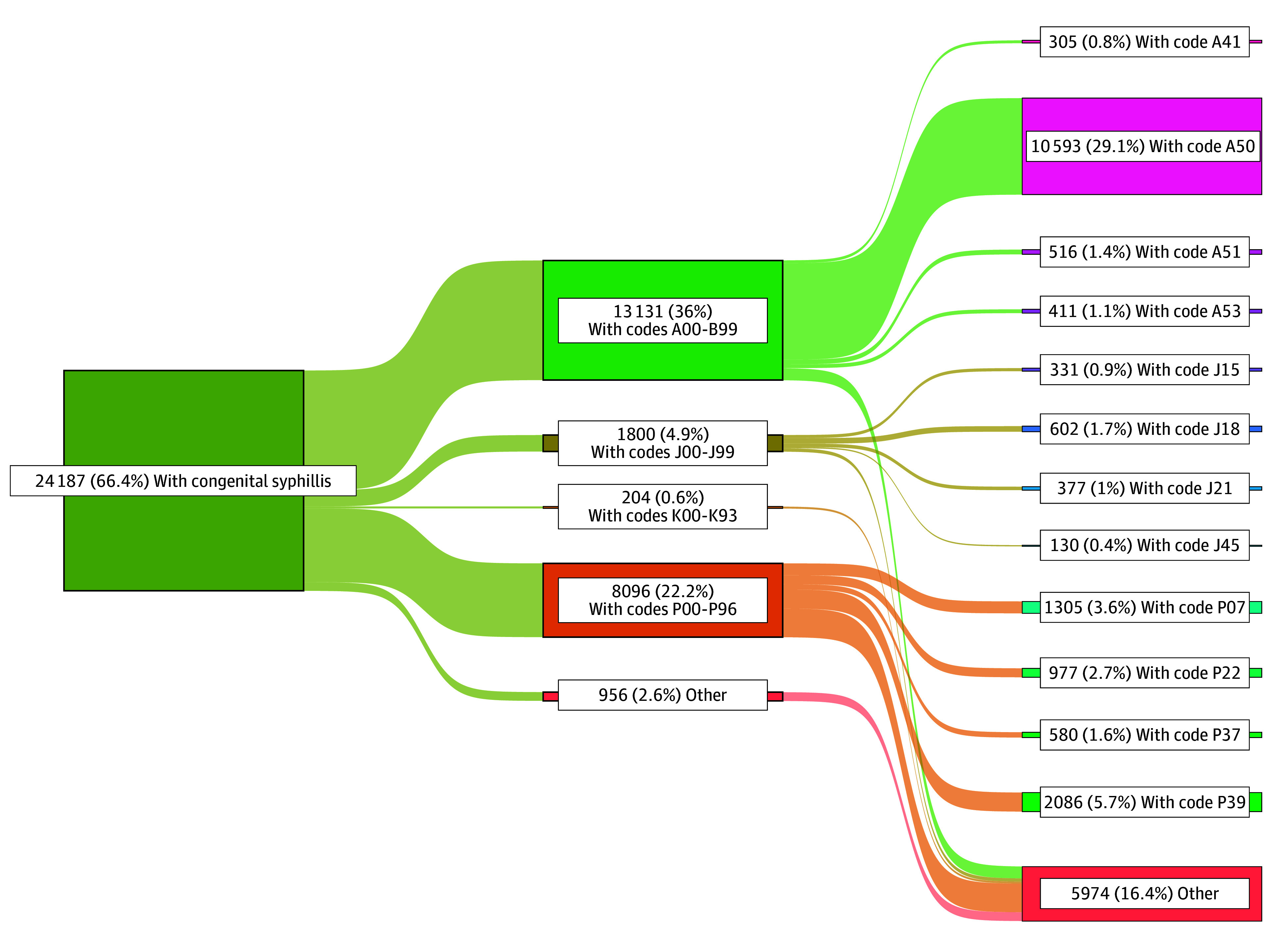
Sankey Plot Showing a Summary of Any ICD-10 Code Specified in the First Hospital for Those With Congenital Syphillis A patient may have more than 1 record during their admission. ICD-10 indicates *International Classification of Diseases and Related Health Problems, Tenth Revision*.

**Figure 4.**
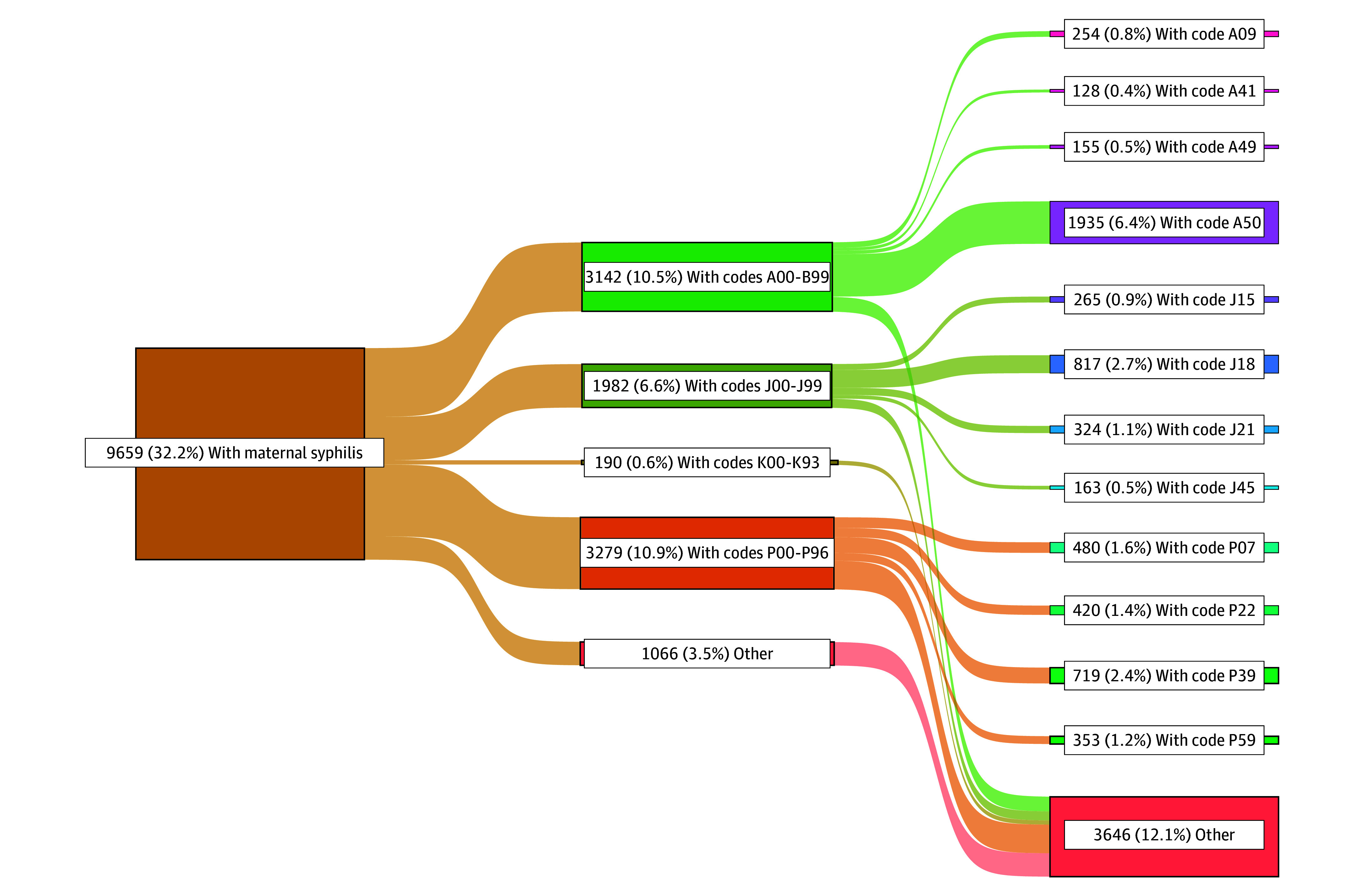
Sankey Plot Showing a Summary of Any ICD-10 Code Specified in the First Hospital for Those Exposed to Maternal Syphilis (Where Congenital Syphilis Was Not Detected at Birth) A patient may have more than 1 record during their admission. ICD-10 indicates *International Classification of Diseases and Related Health Problems, Tenth Revision*.

Figures 3 and 4 display the summary of specific causes of first hospital admission for those exposed to syphilis during pregnancy (nonexposed available in eFigure 2 in Supplement 1). Of the 24 187 live birth records with congenital syphilis who were hospitalized, 13 131 (54.3%) had infectious or parasitic diseases (A00-B99), compared with 3142 of 9659 (32.5%) in the maternal syphilis group and 286 403 of 1 619 758 (17.7%) in the nonexposed group. Perinatal outcomes (P00-P93) occurred in 8096 of 24 187 congenital syphilis records (33.0%), 3279 of 9659 maternal syphilis records (33.9%), and 438 595 of 1 619 758 of nonexposed records (27.1%). Respiratory codes (J00-J99) occurred in 1800 of 24 187 (7.4%), 1982 of 9659 (20.5%), and 547 169 of 1 619 758 (33.8%) records in the congenital, maternal, and nonexposed syphilis groups, respectively. Digestive codes (K00-K93) occurred in 204 of 24 187 congenital syphilis records (0.8%), 190 of 9659 maternal syphilis records (2.0%), and 59 969 of 1 619 758 nonexposed records (3.7%). A50 (congenital syphilis) was the most frequent occurring code for congenital and maternal syphilis, at 10 593 of 24 187 (43.8%) and 1935 of 9659 (20.0%), respectively. For the nonexposed group, the most frequent code was J18 (pneumonia, organism unspecified) at 208 308 of 1 619 758 records (12.9%).

The length of the first hospital admission stay among children with congenital syphilis and maternal syphilis was a mean of 1.64 (95% CI, 1.61-1.68) and 1.22 (95% CI, 1.18-1.27) days longer, respectively, compared with the nonxposed group (eTables 8-10 in Supplement 1).

### Recurrent Hospitalization and Death

Over 5 years of follow-up, those with congenital syphilis were associated with a higher risk of recurrent hospitalization (HR, 1.96; 95% CI, 1.94-1.97) compared with those not exposed (Figure 2; eTables 10-11 in Supplement 1). Similarly, those with maternal syphilis had an increased risk compared with the nonexposed group (HR, 1.37; 95% CI, 1.36-1.39).

During a 5-year follow-up, the risk of death for those with congenital syphilis was higher than for those nonexposed to syphilis (HR, 2.30; 95% CI, 2.15-2.47). Maternal syphilis was associated with an increased risk compared with those nonexposed (HR, 1.29; 95% CI, 1.16-1.44). Any syphilis exposure was associated with an increase in the risk of death (HR, 1.72; 95% CI, 1.62-1.84) (eTables 12-15, eFigure 3 in Supplement 1). For all models, the sensitivity analyses excluding preterm births or low and high birthweight did not change the results (eTables 16-23, eFigure 4 in Supplement 1).

## Discussion

In this population-based cohort study, which included over 8 million live births, syphilis exposure during pregnancy was associated with increased morbidity and mortality risk for those aged under 5 years. Children with congenital syphilis had an over 6-fold increase in the hazard of first hospitalization, while those exposed to maternal syphilis (without congenital syphilis detected at birth) had a nearly 2-fold increase, compared with those not exposed during pregnancy. The highest HR was observed in the first month of life, reaching an 11-fold increase among those with congenital syphilis. Although this decreased with age, children exposed to syphilis continued to have higher hospitalization rates than the nonexposed group until age 3 years. Live-born children exposed to syphilis during pregnancy also had more and longer hospitalizations. Additionally, causes of hospitalizations differed between groups with any syphilis exposure having a higher percentage of perinatal *ICD-10* codes. Those with congenital syphilis had a lower prevalence of respiratory and digestive diagnoses than those with maternal syphilis and the nonexposed group.

The perinatal risks associated with syphilis during pregnancy are well known. However, literature on the long-term morbidity and mortality of children exposed to syphilis during pregnancy is limited, particularly for those exposed to maternal syphilis without congenital syphilis detected at birth. Previous studies focusing on congenital syphilis have shown that live-born children with congenital syphilis have higher mortality rates, especially infants with higher Venereal Disease Research Laboratory (VDRL) test titers and those presenting with signs and symptoms at birth.^9^ Studies conducted in the USA indicated that infants with congenital syphilis had hospital stays 3 times longer and incurred costs 5 times higher than their counterparts without congenital syphilis.^29,30^ Two cohort studies investigated the growth of live-born children exposed to syphilis but uninfected and did not find evidence of impaired growth patterns among these children under 18 months.^31,32^ Our investigation adds to this literature by shedding light on the long-term morbidity of those with congenital syphilis and including the underinvestigated group of those exposed to syphilis but without congenital syphilis detected at birth.

The prenatal period is highly sensitive for the early development of biological systems, with environmental factors playing a crucial role in shaping the growing fetus and the expression of certain disorders.^33^ Therefore, even with highly effective treatment that can prevent most perinatal complications related to syphilis—evidenced by the similar rates of preterm birth between those exposed to maternal syphilis and the nonexposed group—early life exposure and treatment might still lead to long-term outcomes, resulting in the higher hospitalization rates observed in the maternal syphilis group.

### Strengths and Limitations

Our study has several strengths. We used a nationwide linked cohort with a large sample size that can be used to study syphilis and other infectious diseases during pregnancy.^34^ We have included a group of those exposed to syphilis but without congenital infection. We also had a population-representative comparison group and controlled for confounding variables.

Our findings must be interpreted with caution due to limitations. First, as our study used administrative data, relevant clinical data (eg, comparable VDRL titer data for both exposed groups and HIV status among others) were unavailable. However, the general prevalence of HIV among pregnant women in Brazil is approximately 0.4%,^35^ with vertical transmission occurring in about 2 per 100 000 individuals.^36^ Residual confounding may have impacted our estimates; however, the cohort shares comparable socioeconomic conditions as it is based on applications for government social benefits. Additionally, both crude and adjusted measures were similar, suggesting that the potential for residual confounding is minimal.

Misclassification of exposure may occur for several reasons, including Brazil’s sensitive case definition for maternal syphilis, which requires only 1 test (even for asymptomatic women) as a measure to avoid delayed treatment and improve fetal outcomes. This approach may have led to an underestimation of the estimated association. Additionally, the complexity of diagnosing congenital syphilis, which heavily depends on maternal serology and treatment status, may have contributed to misclassification. This is reflected in cases of hospital admissions for congenital syphilis in the exposed group, despite the disease not being detected at birth. Such misclassification could explain the increased risk observed in this group but further research is needed.

Although data quality has been improving over time, poor data can limit the accuracy of data linkage, leading to potential errors. If an error occurred in the linkage indicating exposure (ie, exposure to syphilis) or outcome (eg, hospital admission), it probably led to nondifferential misclassification, and the absolute measures of risk may have been underestimated. The cohort focuses on the most socially vulnerable individuals in Brazil, which has both advantages and limitations. Positively, this minimizes the risk of outcome misclassification, as hospitalization data only captures admission from the Brazilian Unified Health System which is universal and free of charge, and this population is unlikely to have private health insurance or the financial means to cover out of pocket hospital costs. However, this could limit generalizability, although the biological mechanisms underlying these associations are likely applicable to other populations. Third, missing data were present in several variables, including gestational age at birth. While we assumed that missingness was unlikely to be related to the outcome, it is possible that using a complete-case analysis has still potentially introduced bias to our findings. Fourth, while this study has presented results exploring the association between maternal and congenital syphilis and several outcomes, a causal relationship has not been established and is an area for future research. Additionally, since the SIH system was primarily designed for administrative purposes, some entries might refer to the same event, necessitating a threshold to distinguish between 2 different admissions.

## Conclusions

In this cohort study, we found that live-born children exposed to syphilis during pregnancy, even those who were exposed but did not have congenital syphilis detected at birth, were associated with a consistently higher risk of hospital admission and longer lengths of stay during their first 5 years of life compared with those who were not exposed. This study highlights the need for close follow-up and careful monitoring of exposed children. It emphasizes the importance of public health actions not only to reduce vertical transmission but also to prevent acquired syphilis in women of childbearing age before conception. The study results demonstrate the burden of syphilis during pregnancy on the offspring, which can impose substantial costs on families, health care practitioners, and policymakers. This association is particularly pronounced for socially vulnerable groups, who are disproportionately affected by syphilis.

## Notes

Supplement 1. **eTable 1.** Description of datasets **eTable 2.** Baseline characteristics of singleton livebirths from January 1, 2011, to December 31, 2015, by number of admissions **eTable 3.** Baseline characteristics of singleton livebirths from January 1, 2011, to December 31, 2015, by survival status **eTable 4.** Number of events by syphilis status **eTable 5.** Follow-up time to first hospitalization (in months) by syphilis status **eFigure 1**. Kaplan-Meier plot of time, in months, to first hospitalization (all-cause) **eTable 6.** Cox proportional hazards model comparing Syphilis exposure for time with first hospitalization **eTable 7.** Adjusted Cox proportional hazards model comparing Syphilis exposure for time with first hospitalization from a complete-case analysis with time interaction **eFigure 2.** Sankey plot showing a summary of any ICD-10 code specified in the first hospital for those not exposed to syphilis during pregnancy **eTable 8. **Mean (SD) length of stay in first and all hospitalizations **eTable 9.** Gamma regression comparing the mean difference in days of length of stay by syphilis exposure **eTable 10.** Cox proportional hazards model comparing Syphilis exposure for time to readmission **eTable 11.** Adjusted Cox proportional hazards model comparing Syphilis exposure for time to readmission from a complete-case analysis with time interaction **eTable 12.** Number of events by syphilis status **eTable 13.** Follow-up time to death (in months) by syphilis status **eFigure 3.** Kaplan-Meier plot of time to death (in months) **eTable 14.** Cox proportional hazards model comparing Syphilis exposure for time to death **eTable 15.** Adjusted Cox proportional hazards model comparing Syphilis exposure for time to death from a complete-case analysis with time interaction (age in months) **eTable 16.** Adjusted Cox proportional hazards model comparing Syphilis exposure for time to first hospitalization from a complete-case analysis with preterm births excluded **eTable 17.** Adjusted Cox proportional hazards model comparing Syphilis exposure for time to first hospitalization from a complete-case analysis with low (<2500g) and high (≥6,000g) birth weight excluded **eTable 18.** Adjusted gamma regression comparing the mean difference in days of length of stay by syphilis exposure for a complete-case analysis with preterm births excluded **eTable 19.** Adjusted gamma regression comparing the mean difference in days of length of stay by syphilis exposure from a complete-case analysis with low (<2500g) and high (≥6,000g) birth weight excluded **eTable 20.** Adjusted Cox proportional hazards model comparing Syphilis exposure for time to readmission from a complete-case analysis with preterm births excluded **eTable 21.** Adjusted Cox proportional hazards model comparing Syphilis exposure for time to readmission from a complete-case analysis with low (<2500g) and high (≥6,000g) birth weight excluded **eTable 22.** Adjusted Cox proportional hazards model comparing Syphilis exposure for time to death from a complete-case analysis with preterm births excluded **eTable 23.** Adjusted Cox proportional hazards model comparing Syphilis exposure for time to death from a complete-case analysis with low (<2500g) and high (≥6,000g) birth weight excluded **eFigure 4.** Forest plot showing adjusted hazard ratios for (i) the main analysis, (ii) when excluding children with a birth weight under 2,500g or over 6,000g, and (iii) excluding preterm birth (<37 weeks)
eReferences.

Supplement 2.
Data Sharing Statement
