# Supplementary material for: Syphilis Exposure During Pregnancy and Childhood Hospital Admissions in Brazil
Source: JAMA Netw Open. 2025 Apr 30;8(4):e257471. doi: 10.1001/jamanetworkopen.2025.7471 (PMC12044516; doi:10.1001/jamanetworkopen.2025.7471)
Supplement: Supplement 1. — eTable 1. Description of datasets eTable 2. Baseline characteristics of singleton livebirths from January 1, 2011, to December 31, 2015, by number of admissions eTable 3. Baseline characteristics of singleton livebirths from January 1, 2011, to December 31, 2015, by survival status eTable 4. Number of events by syphilis status eTable 5. Follow-up time to first hospitalization (in months) by syphilis status eFigure 1. Kaplan-Meier plot of time, in months, to first hospitalization (all-cause) eTable 6. Cox proportional hazards model comparing Syphilis exposure for time with first hospitalization eTable 7. Adjusted Cox proportional hazards model comparing Syphilis exposure for time with first hospitalization from a complete-case analysis with time interaction eFigure 2. Sankey plot showing a summary of any ICD-10 code specified in the first hospital for those not exposed to syphilis during pregnancy eTable 8. Mean (SD) length of stay in first and all hospitalizations eTable 9. Gamma regression comparing the mean difference in days of length of stay by syphilis exposure eTable 10. Cox proportional hazards model comparing Syphilis exposure for time to readmission eTable 11. Adjusted Cox proportional hazards model comparing Syphilis exposure for time to readmission from a complete-case analysis with time interaction eTable 12. Number of events by syphilis status eTable 13. Follow-up time to death (in months) by syphilis status eFigure 3. Kaplan-Meier plot of time to death (in months) eTable 14. Cox proportional hazards model comparing Syphilis exposure for time to death eTable 15. Adjusted Cox proportional hazards model comparing Syphilis exposure for time to death from a complete-case analysis with time interaction (age in months) eTable 16. Adjusted Cox proportional hazards model comparing Syphilis exposure for time to first hospitalization from a complete-case analysis with preterm births excluded eTable 17. Adjusted Cox proportional hazards model comparing Syphilis ex [file jamanetwopen-e257471-s001.pdf]

Supplemental Online Content

Paixão ES, Carroll O, Rodrigues LC, et al. Syphilis exposure during pregnancy and childhood hospital admissions. *JAMA Netw Open*. 2025;8(4):e257471. doi:10.1001/jamanetworkopen.2025.7471

eTable 1. Description of datasets

eTable 2. Baseline characteristics of singleton livebirths from January 1, 2011, to December 31, 2015, by number of admissions

eTable 3. Baseline characteristics of singleton livebirths from January 1, 2011, to December 31, 2015, by survival status

eTable 4. Number of events by syphilis status

eTable 5. Follow-up time to first hospitalization (in months) by syphilis status

eFigure 1. Kaplan-Meier plot of time, in months, to first hospitalization (all-cause)

eTable 6. Cox proportional hazards model comparing Syphilis exposure for time with first hospitalization

eTable 7. Adjusted Cox proportional hazards model comparing Syphilis exposure for time with first hospitalization from a complete-case analysis with time interaction

eFigure 2. Sankey plot showing a summary of any ICD-10 code specified in the first hospital for those not exposed to syphilis during pregnancy

eTable 8. Mean (SD) length of stay in first and all hospitalizations

eTable 9. Gamma regression comparing the mean difference in days of length of stay by syphilis exposure

eTable 10. Cox proportional hazards model comparing Syphilis exposure for time to readmission

eTable 11. Adjusted Cox proportional hazards model comparing Syphilis exposure for time to readmission from a complete-case analysis with time interaction

eTable 12. Number of events by syphilis status

eTable 13. Follow-up time to death (in months) by syphilis status

eFigure 3. Kaplan-Meier plot of time to death (in months)

eTable 14. Cox proportional hazards model comparing Syphilis exposure for time to death

eTable 15. Adjusted Cox proportional hazards model comparing Syphilis exposure for time to death from a complete-case analysis with time interaction (age in months)

eTable 16. Adjusted Cox proportional hazards model comparing Syphilis exposure for time to first hospitalization from a complete-case analysis with preterm births excluded

© 2025 Paixão ES et al. *JAMA NetworkOpen*.

eTable 17. Adjusted Cox proportional hazards model comparing Syphilis exposure for time to first hospitalization from a complete-case analysis with low (<2500g) and high (≥6,000g) birth weight excluded

eTable 18. Adjusted gamma regression comparing the mean difference in days of length of stay by syphilis exposure for a complete-case analysis with preterm births excluded

eTable 19. Adjusted gamma regression comparing the mean difference in days of length of stay by syphilis exposure from a complete-case analysis with low (<2500g) and high (≥6,000g) birth weight excluded

eTable 20. Adjusted Cox proportional hazards model comparing Syphilis exposure for time to readmission from a complete-case analysis with preterm births excluded

eTable 21. Adjusted Cox proportional hazards model comparing Syphilis exposure for time to readmission from a complete-case analysis with low (<2500g) and high (≥6,000g) birth weight excluded

eTable 22. Adjusted Cox proportional hazards model comparing Syphilis exposure for time to death from a complete-case analysis with preterm births excluded

eTable 23. Adjusted Cox proportional hazards model comparing Syphilis exposure for time to death from a complete-case analysis with low (<2500g) and high (≥6,000g) birth weight excluded

eFigure 4. Forest plot showing adjusted hazard ratios for (i) the main analysis, (ii) when excluding children with a birth weight under 2,500g or over 6,000g, and (iii) excluding preterm birth (<37 weeks)

eReferences.

This supplemental material has been provided by the authors to give readers additional information about their work.

© 2025 Paixão ES et al. *JAMA NetworkOpen*.

1) Study population and Baseline characteristics

1.1) Data description

eTable 1. Description of datasets[1–3]

Data source	Data type	Description of the data and examples of relevant variables
CIDACS Birth Cohort	Live Births<br><br>Social records from CadUnico<br><br>Intersect between live birth linked to social records from individuals whose families applied for social assistance in Brazil	Maternal characteristics (e.g., place of residence, age, marital status, race and skin colour, education, and obstetric history), pregnancy information (e.g., number of prenatal care appointments, gestation age at birth, and type of delivery), characteristics of the live birth (e.g., sex, multiples, birthweight, and presence of congenital anomalies) and housing conditions, urban/rural classification and family composition from CadUnico. This cohort covers over 50% of all birth in the country.
SINAN-syphilis	Information System for Notifiable Diseases (SINAN-Syphilis).	In Brazil, the registration of suspected cases of maternal and congenital syphilis is compulsory. The maternal syphilis form records information about the pregnant person, including the clinical classification of syphilis (e.g., primary, secondary, latent, and tertiary), treatment, and laboratory confirmation. The congenital syphilis form records data on the newborn (e.g., symptoms at birth, treatment, laboratory tests, and treatment) and maternal information (e.g. timing of maternal diagnosis -prenatal, during childbirth or postnatally, and partner treatment).
SIH-SUS	Hospital Information System (SIH).	This system collects information from hospitalizations within the Brazilian Unified Health System (SUS) and includes data on hospital admissions, length of stay, and diagnoses. It has nationwide coverage and is estimated to account for 70% of all hospital admissions in Brazil[12]. For the population we are studying (the population with lower socioeconomic status), it likely reaches much higher coverage.
SIM	Mortality Information System (SIM).	Death-related information are obtained from death certificates. These certificates are legal documents that must be completed by the physician responsible for clinical care, an assistant, or another practitioner from the institution who can attest to the cause of death. This system covers nearly 97% of deaths in Brazil[13].

© 2025 Paixão ES et al. *JAMA NetworkOpen*.

1.2) Baseline characteristics

1.2.1) By hospitalization

eTable 2. Baseline characteristics of singleton livebirths from January 1, 2011, to December 31, 2015, by number of admissions

**Variables (%)**	0<br>(N=6,695,179)	1<br>(N=1,305,093)	2-4<br>(N=264,120)	4+<br>(N=22,475)
Syphilis exposure				
Congenital	12,710 (0.2)	21,818 (1.7)	1,758 (0.7)	157 (0.7)
Maternal	20,651 (0.3)	8,220 (0.6)	1,076 (0.7)	92 (0.4)
Not exposed	6661818 (99.5)	1,285,055 (97.7)	261,286 (98.9)	22,226 (98.9)
Mother's region of residence				
Central-West	2,208,155 (33.0)	98,395 (7.5)	19,414 (7.4)	1,655 (7.4)
North East	2,417,047 (36.1)	448,709 (34.4)	83,836 (31.7)	7,099 (31.6)
North	509,311 (7.6)	152,462 (11.7)	30,080 (11.4)	1,992 (8.9)
South East	2,208,155 (33.0)	450,756 (34.5)	88,762 (33.6)	7,727 (34.4)
South	706,611 (10.6)	154,771 (11.9)	42,028 (15.9)	4,002 (17.8)
Mother's age, years				
<20	1,5600,301 (23.3)	343,367 (26.3)	71,715 (27.2)	6,044 (26.9)
20-34	4,565,874 (68.2)	850,667 (65.2)	173,008 (65.5)	14,523 (64.6)
>=35	568,977 (8.5)	111,056 (8.5)	19,395 (7.3)	1,907 (8.5)
Missing	27 (0.0)	3 (0.0)	2 (0.0)	1 (0.0)
Mean (SD)	24.8 (6.3)	24.4 (6.5)	24.1 (6.3)	24.3 (6.5)
Median (p25, p75)	24.0 (20.0, 29.0)	23.0 (19.0, 29.0)	23.0 (19.0, 28.0)	23.0 (19.0, 28.0)
Mother's education, years				
None	62,049 (0.9)	12,048 (0.9)	2,435 (0.9)	266 (1.2)
1-3	337,398 (5.0)	67,739 (5.2)	14,420 (5.5)	1,367 (6.1)
4-7	1,785,915 (26.7)	386,435 (29.6)	82,483 (31.2)	7,477 (33.3)
8-11	3,957,269 (59.1)	760,454 (58.3)	149,734 (56.7)	12,154 (54.1)
12+	412,833 (6.2)	53,023 (4.1)	9,332 (3.5)	746 (3.3)
Missing	139,715 (2.1)	25,394 (1.9)	5,716 (2.2)	465 (2.1)
Sex				
Female	3,352,296 (50.1)	600,308 (46.0)	112,909 (42.7)	9,339 (41.6)
Male	3,342,055 (49.9)	704,416 (54.0)	151,142 (57.2)	13,117 (58.4)
Missing	828 (0.0)	369 (0.0)	69 (0.0)	19 (0.1)
Mother's Ethnicity				
Asian	1,590,278 (23.8)	297,930 (22.8)	65,721 (24.9)	5,796 (25.8)
Black	362,517 (5.4)	79,995 (6.1)	16,528 (6.3)	1,430 (6.4)
Indigenous	17,239 (0.3)	3,180 (0.2)	604 (0.2)	36 (0.2)
Mixed	3,944,375 (58.9)	762,139 (58.4)	145,882 (55.2)	12,031 (53.5)
White	57,852 (0.9)	9,437 (0.7)	2,702 (1.0)	360 (1.6)
Missing	722,918 (10.8)	152,412 (11.7)	32,683 (12.4)	2,822 (12.6)
Mother's Relationship status				
In relationship	3,423,762 (51.1)	609,672 (46.7)	122,222 (46.3)	10,201 (45.4)
Single[§]	3,179,312 (47.5)	678,778 (52.0)	138,356 (52.4)	11,980 (53.3)

Missing	92,105 (1.4)	16,643 (1.3)	3,542 (1.3)	294 (1.3)
Delivery type				
Caesarean	3,164,686 (47.3)	607,834 (46.6)	127,966 (48.4)	11,353 (50.5)
Vaginal	3,521,728 (52.6)	695,696 (53.3)	135,770 (51.4)	11,096 (49.4)
Missing	8,765 (0.1)	1,563 (0.1)	384 (0.1)	26 (0.1)
Birth weight, grams				
<1500	32,534 (0.5)	38,755 (3.0)	4,736 (1.8)	781 (3.5)
1500-2499	301,586 (4.5)	158,262 (12.1)	24,216 (9.2)	2,610 (11.6)
2500-5999	6,355,168 (94.9)	1,107,528 (84.9)	235,088 (89.0)	19,079 (84.9)
>=6000	194 (0.0)	45 (0.0)	8 (0.0)	0 (0.0)
Missing	5,697 (0.1)	503 (0.0)	72 (0.0)	5 (0.0)
Mean (SD)	3,246.4 (491.3)	3,078.6 (662.7)	3,135.1 (585.9)	3,039.9 (649.5)
Median (p25, p75)	3,250.0 (2,956.0, 3,550.0)	3,150.0 (2,770.0, 3,500.0)	3,180 (2,845.0, 3,500.0)	3,100.0 (2740.0, 3,450.0)
Gestational age at birth, weeks				
18-31	50,716 (0.8)	39,661 (3.0)	5,096 (1.9)	767 (3.4)
32-36	480,248 (7.2)	175,918 (13.5)	28,330 (10.7)	2,774 (12.3)
37-44	5,429,518 (81.1)	934,152 (71.6)	195,870 (74.2)	15,912 (70.8)
Mean (SD)	38.8 (2.1)	38.1 (2.8)	38.4 (2.5)	38.1 (2.9)
Median (p25, p75)	39.0 (38.0, 40.0)	39.0 (37.0, 40.0)	39.0 (38.0, 40.0)	39.0 (37.0, 40.0)
Number of prenatal appointments				
0	176,489 (2.6)	41,389 (3.2)	7,035 (2.7)	667 (3.0)
1-3	539,309 (8.1)	128,555 (9.9)	22,529 (8.5)	2,061 (9.2)
4-6	1,995,234 (29.8)	420,554 (32.2)	80,678 (30.5)	6,810 (30.3)
7+	3,961,906 (58.7)	701,296 (53.7)	151,743 (57.5)	12,740 (56.7)
Missing	52,241 (0.8)	13,299 (1.0)	2,135 (0.8)	197 (0.9)
Mean (SD)	7.1 (2.8)	6.9 (2.9)	7.1 (2.9)	7.1 (3.0)
Median (p25, p75)	7.0 (5.0, 9.0)	7.0 (5.0, 9.0)	7.0 (5.0, 9.0)	7.0 (5.0, 9.0)
Year of birth				
2011	1,212,182 (18.1)	290,981 (22.3)	61,652 (23.3)	5,316 (23.7)
2012	1,250,168 (18.7)	281,855 (21.6)	59,804 (22.6)	5,165 (23.0)
2013	1,379,879 (20.6)	254,302 (19.5)	52,760 (20.0)	4,598 (20.5)
2014	1,466,826 (21.9)	253,011 (19.4)	50,074 (19.0)	4,341 (19.3)
2015	1,386,124 (20.7)	224,944 (17.2)	39,830 (15.1)	3,055 (13.6)

§ Single refers to those who are not in a relationship, separated, divorced or widowed.
p25, p75 are the 25th and 75th percentiles. SD: standard deviation

© 2025 Paixão ES et al. *JAMA NetworkOpen*.

1.2.2) By Death

eTable 3. Baseline characteristics of singleton livebirths from January 1, 2011, to December 31, 2015, by survival status

**Variables (%)**	Alive (N=8,200,250)	Died (N=86,617)
Syphilis exposure		
Congenital	35,525 (0.4)	918 (1.1)
Maternal	29,623 (0.4)	416 (0.5)
Not exposed	8,135,102 (99.2)	85,283 (98.5)
Mother's region of residence		
Central-West	622,251 (7.6)	6,524 (7.5)
North East	2,924,520 (35.7)	32,171 (37.1)
North	1,026,285 (12.5)	12,304 (14.2)
South East	2,728,044 (33.3)	27,356 (31.6)
South	899,150 (11.0)	8,262 (9.5)
Mother's age, years		
<20	1,956,977 (23.9)	24,450 (28.2)
20-34	5,550,863 (67.7)	53,209 (61.4)
>=35	692,379 (8.4)	8956 (10.3)
Missing	31 (0.0)	2 (0.0)
Mean (SD)	24.7 (6.4)	24.5 (6.9)
Median (p25, p75)	24.0 (20.0, 29.0)	23.0 (19.0, 29.0)
Mother's education, years		
None	75,195 (0.9)	1,603 (1.9)
1-3	414,806 (5.1)	6,118 (7.1)
4-7	2,234,761 (27.3)	27,549 (31.8)
8-11	4,833,925 (58.9)	45,686 (52.7)
12+	472,350 (5.8)	3,584 (4.1)
Missing	169,213 (2.1)	2,077 (2.4)
Sex		
Female	4,036,499 (49.2)	38,353 (44.3)
Male	4,162,560 (50.8)	48,170 (55.6)
Missing	1,191 (0.0)	94 (0.1)
Mother's Ethnicity		
Asian	1,942,177 (23.7)	17,548 (20.3)
Black	454,871 (5.5)	5,599 (6.5)
Indigenous	20,849 (0.3)	210 (0.2)
Mixed	4,812,776 (58.7)	51,651 (59.6)
White	68,831 (0.8)	1,520 (1.8)
Missing	900,746 (11.0)	10,089 (11.6)
Mother's Relationship status		
In relationship	4,126,133 (50.3)	39,724 (45.9)
Single[§]	3,962,908 (48.3)	45,518 (52.6)
Missing	111,209 (1.4)	1,375 (1.6)
Delivery type		

© 2025 Paixão ES et al. *JAMA NetworkOpen*.

eTable 3. Baseline characteristics of singleton livebirths from January 1, 2011, to December 31, 2015, by survival status

**Variables (%)**	Alive (N=8,200,250)	Died (N=86,617)
Caesarean	3,874,623 (47.3)	37,216 (43.0)
Vaginal	4,315,020 (52.6)	49,270 (56.9)
Missing	10,607 (0.1)	131 (0.2)
Birth weight, grams		
<1500	50,405 (0.6)	26,401 (30.5)
1500-2499	470,211 (5.7)	16,463 (19.0)
2500-5999	7,673,314 (93.6)	43,549 (50.3)
>=6000	231 (0.0)	16 (0.0)
Missing	6,089 (0.1)	188 (0.2)
Mean (SD)	3,225.8 (510.7)	2,269.7 (1,105.0)
Median (p25, p75)	3,235.0 (2,935.0, 3,540.0)	2,500 (1,190.0, 3,160.0)
Gestational age at birth, weeks		
18-31	74,316 (0.9)	21,924 (25.3)
32-36	673,380 (8.2)	13,890 (16.0)
37-44	6,536,637 (79.7)	38,815 (44.8)
Missing	897,256 (10.9)	11,837 (13.7)
Mean (SD)	38.7 (2.1)	34.5 (6.0)
Median (p25, p75)	39.0 (38.0, 40.0)	37.0 (30.0, 39.0)
Number of prenatal appointments		
0	218,713 (2.7)	6,867 (7.9)
1-3	674,474 (8.2)	17,980 (20.8)
4-6	2,472,709 (30.2)	30,567 (35.3)
7+	4,768,459 (58.2)	29,226 (33.7)
Missing	65,895 (0.8)	1,977 (2.3)
Mean (SD)	7.1 (2.8)	5.6 (3.1)
Median (p25, p75)	7.0 (5.0, 9.0)	5.0 (3.0, 7.0)
Year of birth		
2011	1,551,961 (18.9)	18,170 (21.0)
2012	1,579,286 (19.3)	17,706 (20.4)
2013	1,674,153 (20.4)	17,386 (20.1)
2014	1,756,955 (21.4)	17,297 (20.0)
2015	1,637,895 (20.0)	16,058 (18.5)

§ Single refers to those who are not in a relationship, separated, divorced or widowed.
p25, p75 are the 25th and 75th percentiles. SD: standard deviation

© 2025 Paixão ES et al. *JAMA NetworkOpen*.

2) Analyses

2.1) Time to first hospitalization

eTable 4. Number of events by syphilis status

**Event (%)**	**Congenital (N=36,443)**	**Maternal (N=30,039)**	**Not Exposed (N=8,220,385)**
Not hospitalised	12,710 (34.9)	20,651 (68.7)	6,661,818 (81.0)
First hospitalization	23,733 (65.1)	9,388 (31.3)	1,558,567 (19.0)

eTable 5. Follow-up time to first hospitalization (in months) by syphilis status

**Follow-up time, months**	**Congenital (N=33,305)**	**Maternal (N=25,986)**	**Not Exposed (N=7,233,991)**
Mean (SD)	19.6 (25.6)	38.2 (23.7)	46.7 (19.2)
Median (p25, p75)	0.1 (0.0, 48.0)	47.0 (10.5, 60.0)	57.5 (41.2, 60.0)

p25, p75 are the 25th and 75th percentiles. SD: standard deviation

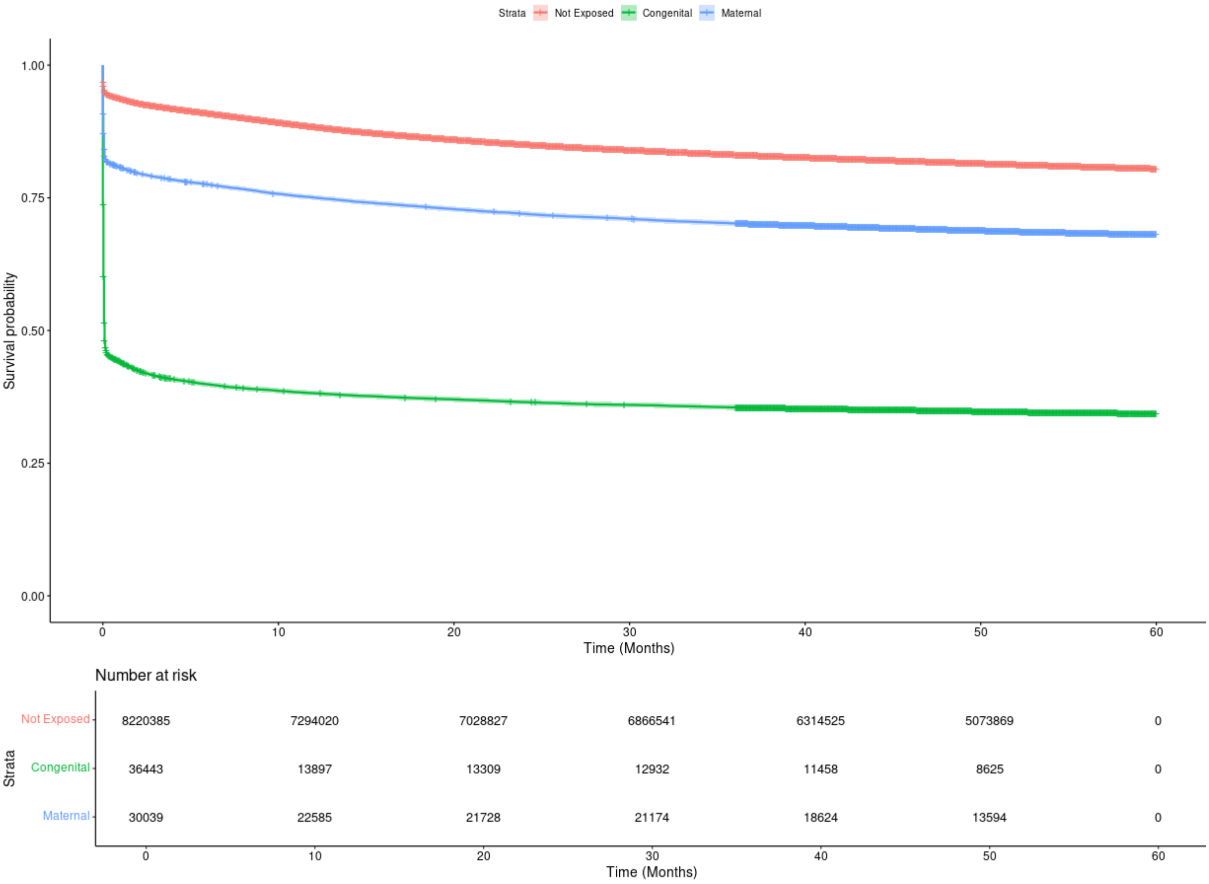

eFigure 1. Kaplan-Meier plot of time, in months, to first hospitalization (all-cause)
Red: Not exposed to syphilis during pregnancy; Blue: Maternal syphilis; Green: Congenital syphilis.

© 2025 Paixão ES et al. *JAMA NetworkOpen*.

eTable 6. Cox proportional hazards model comparing Syphilis exposure for time with first hospitalization. Adjusted analysis uses a complete-case analysis.

**Exposure status**		**Hazard ratio**	**95% Confidence Interval**	
Unadjusted Cox model (N=8,286,867)				
Congenital vs Not Exposed		6.49	6.41	6.57
Maternal vs Not Exposed		1.90	1.86	1.94
Congenital or Maternal vs Not Exposed		3.51	3.47	3.56
Adjusted Cox model (N=7,200,719)				
Congenital vs Not Exposed		6.19	6.11	6.28
Maternal vs Not Exposed		1.90	1.86	1.94
Congenital or Maternal vs Not Exposed		3.43	3.39	3.47
Region	North-East	0.88	0.88	0.89
	North	0.84	0.84	0.85
	Central-West	0.93	0.93	0.94
	South	1.18	1.17	1.19
Mother's Education	1-3 years	0.98	0.97	1.00
	4-7 years	1.02	1.00	1.04
	8- 11 years	0.93	0.92	0.95
	12+ years	0.66	0.65	0.67
Year of birth	2012	0.91	0.90	0.91
	2013	0.76	0.76	0.77
	2014	0.74	0.73	0.74
	2015	0.74	0.73	0.74
Age of mother		0.99	0.99	0.99
Relationship status	Single[§]	1.11	1.11	1.12
Ethnicity	Asian	0.98	0.96	1.00
	Black	1.13	1.11	1.16
	Indigenous	1.01	0.97	1.04
	Mixed	1.06	1.04	1.08

[§] Single refers to those who are not in a relationship, separated, divorced or widowed.

© 2025 Paixão ES et al. *JAMA NetworkOpen*.

eTable 7. Adjusted Cox proportional hazards model comparing Syphilis exposure for time with first hospitalization from a complete-case analysis with time interaction (age in months) (N= 36,806,990 records; 7,200,719 patients).

**Exposure status**		**Hazard ratio**	**95% Confidence Interval**	
Exposure by age (months)				
Congenital vs Not Exposed	1	11.53	11.36	11.70
	12	2.37	2.26	2.47
	24	1.13	1.04	1.23
	36	1.12	1.00	1.25
	48	1.03	0.89	1.19
	60	0.95	0.78	1.15
Maternal vs Not Exposed	1	3.28	3.19	3.73
	12	1.24	1.17	1.30
	24	1.06	0.99	1.14
	36	1.06	0.97	1.16
	48	0.98	0.86	1.10
	60	0.92	0.78	1.08
Congenital or Maternal vs Not Exposed	1	6.15	6.06	6.25
	12	1.71	1.65	1.77
	24	1.09	1.03	1.15
	36	1.09	1.01	1.17
	48	1.00	0.91	1.10
	60	0.93	0.82	1.06
Adjusted variables				
Region	North-East	0.88	0.88	0.89
	North	0.84	0.84	0.85
	Central-West	0.93	0.93	0.94
	South	1.18	1.17	1.19
Mother's Education	1-3 years	0.98	0.97	1.00
	4-7 years	1.02	1.00	1.04
	8- 11 years	0.93	0.92	0.95
	12+ years	0.66	0.65	0.67
Year of birth	2012	0.91	0.90	0.91
	2013	0.76	0.76	0.77
	2014	0.74	0.73	0.74
	2015	0.74	0.73	0.74

© 2025 Paixão ES et al. *JAMA NetworkOpen*.

Exposure status		Hazard ratio	95% Confidence Interval	
Age of mother		0.99	0.99	0.99
Relationship status	Single[§]	1.11	1.10	1.11
Ethnicity	Asian	0.98	0.96	1.00
	Black	1.13	1.11	1.16
	Indigenous	1.01	0.97	1.04
	Mixed	1.06	1.04	1.08

[§] Single refers to those who are not in a relationship, separated, divorced or widowed.

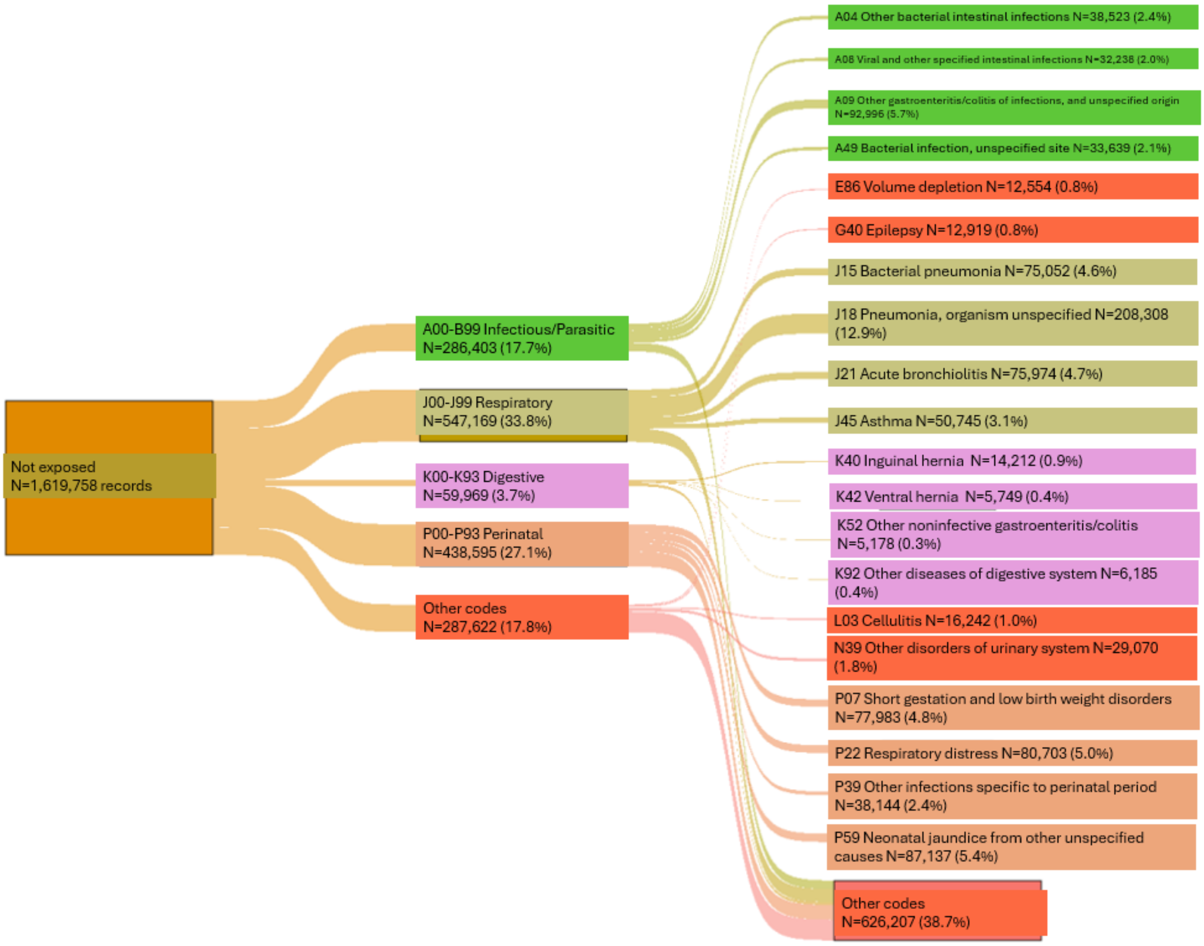

eFigure 2. Sankey plot showing a summary of any ICD-10 code specified in the first hospital for those not exposed to syphilis during pregnancy.

A patient may have more than one record during their admission.

© 2025 Paixão ES et al. *JAMA NetworkOpen*.

2.2) Length of Stay

eTable 8. Mean (SD) length of stay in first and all hospitalizations

	**Congenital (N= 21,732)**	**Maternal (N= 8,214)**	**Not Exposed (N= 1,368,534)**
First hospitalization	11.3 (10.6)	8.4 (10.5)	6.6 (11.1)
All hospitalizations	12.3 (13.4)	9.7 (13.9)	8.4 (16.2)

eTable 9. Gamma regression comparing the mean difference in days of length of stay by syphilis exposure. Adjusted results use a complete-case analysis.

**Exposure status**		**Mean difference**	**95% Confidence Interval**	
Unadjusted (N=1,591,688)				
Congenital vs Not Exposed		1.72	1.68	1.76
Maternal vs Not Exposed		1.28	1.24	1.32
Congenital or Maternal vs Not Exposed		1.48	1.45	1.51
Adjusted (N=1,371,307)				
Congenital vs Not Exposed		1.64	1.61	1.68
Maternal vs Not Exposed		1.22	1.18	1.27
Congenital or Maternal vs Not Exposed		1.42	1.39	1.45
Region	North-East	0.85	0.85	0.86
	North	0.81	0.80	0.81
	Central-West	0.85	0.84	0.86
	South	0.96	0.96	0.97
Mother's Education	1-3 years	0.93	0.90	0.96
	4-7 years	0.92	0.90	0.95
	8- 11 years	0.91	0.88	0.93
	12+ years	0.90	0.87	0.93
Year of birth	2012	1.01	1.00	1.02
	2013	1.05	1.04	1.06
	2014	1.07	1.06	1.08
	2015	1.10	1.09	1.11
Age of mother		1.01	1.01	1.01
Relationship status	Single[§]	1.06	1.05	1.06
Ethnicity	Asian	0.99	0.96	1.02
	Black	1.01	0.98	1.04
	Indigenous	0.97	0.91	1.03
	Mixed	0.99	0.96	1.02

[§] Single refers to those who are not in a relationship, separated, divorced or widowed.

© 2025 Paixão ES et al. *JAMA NetworkOpen*.

2.3) Time to readmission

eTable 10. Cox proportional hazards model comparing Syphilis exposure for time to readmission. Adjusted results are from a complete-case analysis.

**Exposure status**		**Hazard ratio**	**95% Confidence Interval**	
Unadjusted (N=12,192,551 records)				
Congenital vs Not Exposed		2.49	2.47	2.50
Maternal vs Not Exposed		1.47	1.44	1.49
Congenital or Maternal vs Not Exposed		1.91	1.89	1.92
Adjusted (N=10,559,899 records)				
Congenital vs Not Exposed		1.96	1.94	1.97
Maternal vs Not Exposed		1.37	1.36	1.39
Congenital or Maternal vs Not Exposed		1.64	1.63	1.65
Region	North-East	0.97	0.96	0.97
	North	0.94	0.94	0.95
	South-East	1.03	1.02	1.03
	South	1.12	1.11	1.12
Mother's Education	1-3 years	1.00	0.99	1.01
	4-7 years	1.02	1.01	1.03
	8- 11 years	0.97	0.96	0.98
	12+ years	0.78	0.77	0.79
Year of birth	2012	0.95	0.95	0.95
	2013	0.86	0.86	0.87
	2014	0.84	0.84	0.85
	2015	0.84	0.83	0.84
Age of mother		1.00	1.00	1.00
Relationship status	Single[§]	1.06	1.06	1.06
Ethnicity	Asian	1.00	0.99	1.01
	Black	1.08	1.07	1.09
	Indigenous	1.01	0.99	1.03
	Mixed	1.05	1.03	1.06
Previous event		1.63	1.63	1.63

[§] Single refers to those who are not in a relationship, separated, divorced or widowed.

© 2025 Paixão ES et al. *JAMA NetworkOpen*.

eTable 11. Adjusted Cox proportional hazards model comparing Syphilis exposure for time to readmission from a complete-case analysis with time interaction (age in months) (N= 62,207,645 records).

Exposure status		Hazard ratio	95% Confidence Interval	
Exposure by age (months)				
Congenital vs Not Exposed	1	3.58	2.51	3.64
	12	2.02	1.99	2.04
	24	3.70	3.58	3.83
	36	2.07	1.93	2.21
	48	1.92	1.77	2.09
	60	1.82	1.65	2.00
Maternal vs Not Exposed	1	2.02	1.96	2.09
	12	1.45	1.43	1.48
	24	1.76	1.66	1.88
	36	1.35	1.22	1.49
	48	1.28	1.13	1.45
	60	1.27	1.11	1.46
Congenital or Maternal vs Not Exposed	1	2.69	2.64	2.74
	12	1.71	1.69	1.73
	24	2.56	2.47	2.64
	36	1.67	1.57	1.77
	48	1.57	1.46	1.69
	60	1.52	1.40	1.65
Adjusted variables				
Region	North-East	0.97	0.96	0.97
	North	0.94	0.94	0.95
	South-East	1.03	1.02	1.03
	South	1.12	1.11	1.12
Mother's Education	1-3 years	1.00	0.99	1.01
	4-7 years	1.02	1.01	1.03
	8- 11 years	0.97	0.96	0.98
	12+ years	0.78	0.77	0.79
Year of birth	2012	0..95	0.95	0.95
	2013	0.86	0.86	0.87
	2014	0.84	0.84	0.85
	2015	0.84	0.83	0.84

© 2025 Paixão ES et al. *JAMA NetworkOpen*.

Exposure status		Hazard ratio	95% Confidence Interval	
Age of mother		1.00	1.00	1.00
Relationship status	Single[§]	1.06	1.06	1.06
Ethnicity	Asian	1.00	0.99	1.01
	Black	1.08	1.07	1.09
	Indigenous	1.01	0.99	1.03
	Mixed	1.05	1.03	1.06

[§] Single refers to those who are not in a relationship, separated, divorced or widowed.

© 2025 Paixão ES et al. *JAMA NetworkOpen*.

2.4) Time to death

eTable 12. Number of events by syphilis status

Event (%)	Congenital (N=36,443)	Maternal (N=30,039)	Not Exposed (N=8,220,385)
Alive	35,536 (97.5)	29,635 (98.7)	8,137,250 (99.0)
Died	907 (2.5)	404 (1.3)	83,135 (1.0)

eTable 13. Follow-up time to death (in months) by syphilis status

Follow-up time	Congenital (N=36,443)	Maternal (N=30,039)	Not Exposed (N=8,220,385)
Mean (SD)	19.6 (25.6)	38.2 (23.7)	46.7 (19.2)
Median (p25, p75)	0.1 (0.0, 48.0)	47.0 (10.5, 60.0)	57.5 (41.2, 60.0)

p25, p75 are the 25th and 75th percentiles. SD: standard deviation

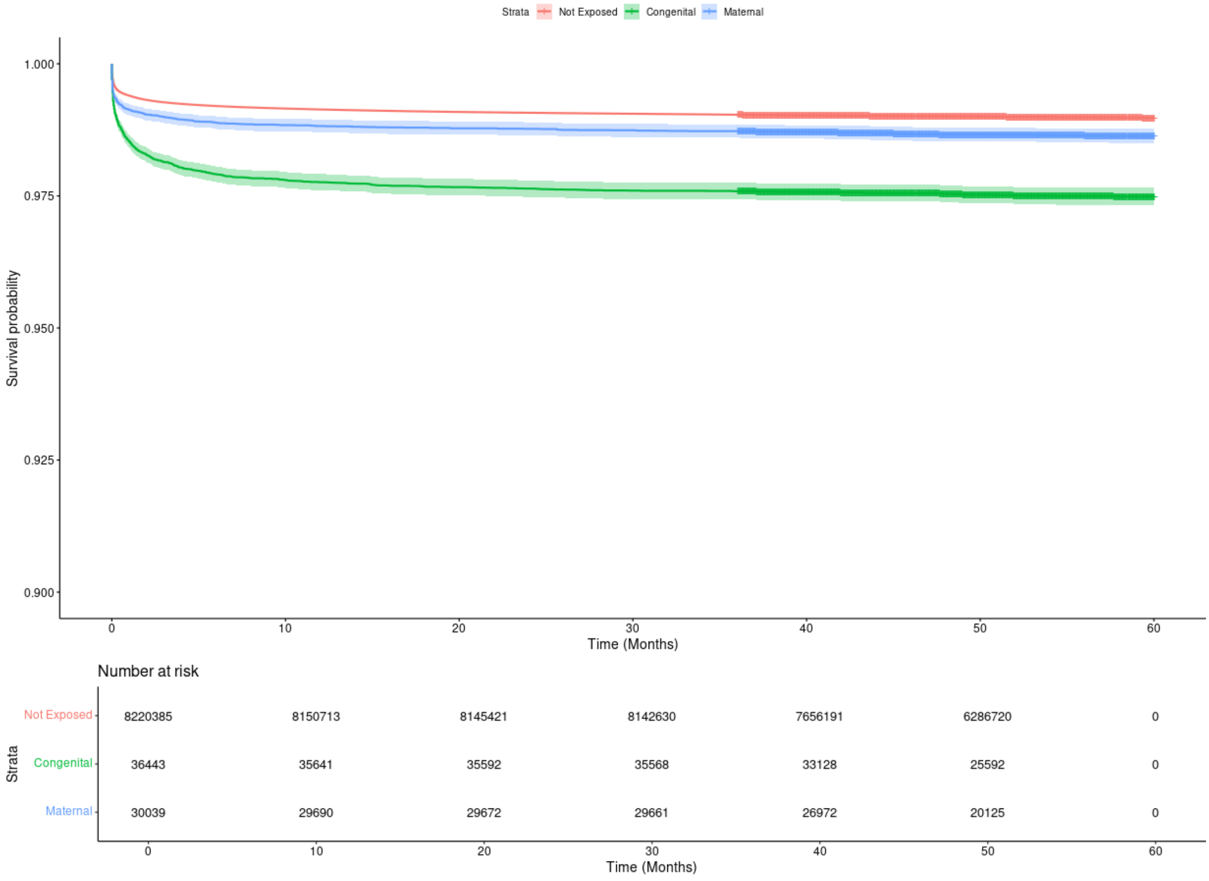

eFigure 3. Kaplan-Meier plot of time to death (in months)

© 2025 Paixão ES et al. *JAMA NetworkOpen*.

eTable 14. Cox proportional hazards model comparing Syphilis exposure for time to death. Adjusted results are from a complete-case analysis.

**Exposure status**		**Hazard ratio**	**95% Confidence Interval**	
Unadjusted (N=8,286,867 patients)				
Congenital vs Not Exposed		2.49	2.33	2.65
Maternal vs Not Exposed		1.34	1.21	1.48
Congenital or Maternal vs Not Exposed		1.82	1.72	1.93
Adjusted (N=7,200,719 patients)				
Congenital vs Not Exposed		2.30	2.15	2.47
Maternal vs Not Exposed		1.29	1.16	1.44
Congenital or Maternal vs Not Exposed		1.72	1.62	1.84
Region	North-East	1.10	1.07	1.13
	North	1.23	1.19	1.28
	Central-West	1.08	1.03	1.13
	South	0.97	0.93	1.01
Mother's Education	1-3 years	0.71	0.66	0.77
	4-7 years	0.55	0.51	0.59
	8- 11 years	0.39	0.36	0.42
	12+ years	0.30	0.27	0.33
Year of birth	2012	0.98	0.95	1.02
	2013	0.95	0.92	0.99
	2014	0.97	0.93	1.00
	2015	1.00	0.97	1.04
Age of mother		1.00	1.00	1.00
Relationship status	Single[§]	1.13	1.10	1.15
Ethnicity	Asian	0.39	0.36	0.42
	Black	0.52	0.48	0.57
	Indigenous	0.51	0.41	0.62
	Mixed	0.45	0.42	0.48

[§] Single refers to those who are not in a relationship, separated, divorced or widowed.

© 2025 Paixão ES et al. *JAMA NetworkOpen*.

eTable 15. Adjusted Cox proportional hazards model comparing Syphilis exposure for time to death from a complete-case analysis with time interaction (age in months) (N= 36,806,990 records, 7,200,719 patients).

Exposure status		Hazard ratio	95% Confidence Interval	
Exposure by age (months)				
Congenital vs Not Exposed	1	2.22	2.03	2.44
	12	2.85	2.52	3.22
	24	1.88	1.38	2.56
	36	1.14	0.69	1.90
	48	1.42	0.82	2.44
	60	2.07	1.20	3.57
Maternal vs Not Exposed	1	1.39	1.22	1.59
	12	1.20	0.97	1.49
	24	0.65	0.36	1.67
	36	1.09	0.59	1.94
	48	2.00	1.18	3.38
	60	1.09	0.45	2.63
Congenital or Maternal vs Not Exposed	1	1.75	1.62	1.91
	12	1.85	1.64	2.09
	24	1.10	0.79	1.54
	36	1.11	0.75	1.63
	48	1.68	1.15	2.46
	60	1.50	0.89	2.52
Adjusted variables				
Region	North-East	1.05	1.03	1.07
	North	1.12	1.09	1.15
	Central-West	1.02	0.99	1.05
	South	0.94	0.91	0.97
Mother's Education	1-3 years	0.74	0.70	0.78
	4-7 years	0.62	0.59	0.66
	8- 11 years	0.50	0.47	0.52
	12+ years	0.42	0.39	0.44
Year of birth	2012	0.98	0.96	1.00
	2013	0.95	0.92	0.97
	2014	0.94	0.91	0.96
	2015	0.96	0.94	1.00

© 2025 Paixão ES et al. *JAMA NetworkOpen*.

Exposure status		Hazard ratio	95% Confidence Interval	
Age of mother		1.00	0.99	1.00
Relationship status	Single[§]	1.16	1.15	1.18
Ethnicity	Asian	0.54	0.51	0.57
	Black	0.66	0.62	0.70
	Indigenous	0.57	0.49	0.66
	Mixed	0.58	0.55	0.61

[§] Single refers to those who are not in a relationship, separated, divorced or widowed.

© 2025 Paixão ES et al. *JAMA NetworkOpen*.

3) Supplementary Analyses

3.1) Time to first hospitalization

eTable 16. Adjusted Cox proportional hazards model comparing Syphilis exposure for time to first hospitalization from a complete-case analysis with preterm births excluded (N= 6,197,689).

Exposure status		Hazard ratio	95% Confidence Interval	
Exposure				
Congenital vs Not Exposed		6.70	6.60	6.80
Maternal vs Not Exposed		1.98	1.94	2.03
Congenital or Maternal vs Not Exposed		3.64	3.59	3.70
Adjusted variables				
Region	North-East	0.91	0.90	0.91
	North	0.89	0.88	0.89
	Central-West	0.95	0.94	0.96
	South	1.18	1.18	1.19
Mother's Education	1-3 years	0.97	0.95	0.99
	4-7 years	1.00	0.98	1.02
	8- 11 years	0.91	0.89	0.93
	12+ years	0.64	0.62	0.65
Year of birth	2012	0.90	0.89	0.90
	2013	0.75	0.75	0.76
	2014	0.73	0.72	0.73
	2015	0.72	0.72	0.73
Age of mother		0.99	0.99	0.99
Relationship status	Single[§]	1.11	1.10	1.11
Ethnicity	Asian	0.94	0.92	0.96
	Black	1.10	1.08	1.13
	Indigenous	0.96	0.92	1.01
	Mixed	1.03	1.01	1.05

[§] Single refers to those who are not in a relationship, separated, divorced or widowed.

© 2025 Paixão ES et al. *JAMA NetworkOpen*.

eTable 17. Adjusted Cox proportional hazards model comparing Syphilis exposure for time to first hospitalization from a complete-case analysis with low (<2500g) and high (≥6,000g) birth weight excluded (N= 6,709,819).

**Exposure status**		**Hazard ratio**	**95% Confidence Interval**	
Exposure				
Congenital vs Not Exposed		6.59	6.49	6.69
Maternal vs Not Exposed		1.94	1.90	1.99
Congenital or Maternal vs Not Exposed		3.57	3.53	3.63
Adjusted variables				
Region	North-East	0.90	0.90	0.91
	North	0.88	0.88	0.89
	Central-West	0.95	0.95	0.96
	South	1.19	1.18	1.20
Mother's Education	1-3 years	0.98	0.96	1.00
	4-7 years	1.02	1.00	1.04
	8- 11 years	0.94	0.92	0.95
	12+ years	0.66	0.65	0.67
Year of birth	2012	0.91	0.90	0.91
	2013	0.76	0.75	0.76
	2014	0.73	0.73	0.74
	2015	0.73	0.73	0.74
Age of mother		0.99	0.99	0.99
Relationship status	Single[§]	1.10	1.10	1.11
Ethnicity	Asian	0.93	0.91	0.95
	Black	1.08	1.06	1.10
	Indigenous	0.96	0.92	1.00
	Mixed	1.01	0.99	1.03

[§] Single refers to those who are not in a relationship, separated, divorced or widowed.

© 2025 Paixão ES et al. *JAMA NetworkOpen*.

3.2) Length of stay

eTable 18. Adjusted gamma regression comparing the mean difference in days of length of stay by syphilis exposure for a complete-case analysis with preterm births excluded (N= 1,083,019).

Exposure status		Mean difference	95% Confidence Interval	
Exposure				
Congenital vs Not Exposed		1.78	1.74	1.83
Maternal vs Not Exposed		1.31	1.26	1.36
Congenital or Maternal vs Not Exposed		1.53	1.49	1.56
Adjusted variables				
Region	North-East	0.90	0.89	0.90
	North	0.88	0.87	0.89
	Central-West	0.88	0.87	0.89
	South	0.95	0.94	0.96
Mother's Education	1-3 years	0.94	0.91	0.97
	4-7 years	0.91	0.89	0.95
	8- 11 years	0.88	0.85	0.91
	12+ years	0.83	0.80	0.86
Year of birth	2012	1.01	1.00	1.02
	2013	1.04	1.03	1.05
	2014	1.06	1.05	1.07
	2015	1.08	1.07	1.09
Age of mother		1.01	1.00	1.01
Relationship status	Single§	1.05	1.05	1.06
Ethnicity	Asian	0.89	0.86	0.92
	Black	0.94	0.91	0.97
	Indigenous	0.91	0.86	0.98
	Mixed	0.92	0.89	0.95

§ Single refers to those who are not in a relationship, separated, divorced or widowed.

© 2025 Paixão ES et al. *JAMA NetworkOpen*.

eTable 19. Adjusted gamma regression comparing the mean difference in days of length of stay by syphilis exposure from a complete-case analysis with low (<2500g) and high (≥6,000g) birth weight excluded (N= 1,174,047).

**Exposure status**		**Mean difference**	**95% Confidence Interval**	
Exposure				
Congenital vs Not Exposed		1.79	1.75	1.83
Maternal vs Not Exposed		1.31	1.26	1.35
Congenital or Maternal vs Not Exposed		1.53	1.50	1.56
Adjusted variables				
Region	North-East	0.91	0.90	0.91
	North	0.89	0.88	0.90
	Central-West	0.88	0.87	0.89
	South	0.96	0.95	0.97
Mother's Education	1-3 years	0.94	0.91	0.97
	4-7 years	0.92	0.90	0.95
	8- 11 years	0.89	0.86	0.91
	12+ years	0.84	0.81	0.87
Year of birth	2012	1.01	1.00	1.02
	2013	1.04	1.03	1.05
	2014	1.06	1.05	1.07
	2015	1.08	1.07	1.09
Age of mother		1.01	1.00	1.01
Relationship status	Single[§]	1.05	1.04	1.06
Ethnicity	Asian	0.88	0.86	0.91
	Black	0.92	0.90	0.95
	Indigenous	0.86	0.81	0.92
	Mixed	0.91	0.88	0.94

[§] Single refers to those who are not in a relationship, separated, divorced or widowed.

© 2025 Paixão ES et al. *JAMA NetworkOpen*.

3.3) Time to readmission

eTable 20. Adjusted Cox proportional hazards model comparing Syphilis exposure for time to readmission from a complete-case analysis with preterm births excluded (N= 8,868,505 records).

Exposure status		Hazard ratio	95% Confidence Interval	
Exposure				
Congenital vs Not Exposed		2.05	2.03	2.07
Maternal vs Not Exposed		1.41	1.39	1.43
Congenital or Maternal vs Not Exposed		1.70	1.69	1.71
Adjusted variables				
Region	North-East	0.97	0.97	0.98
	North	0.96	0.96	0.97
	South-East	1.02	1.01	1.02
	South	1.11	1.11	1.12
Mother's Education	1-3 years	0.99	0.98	1.00
	4-7 years	1.01	0.99	1.02
	8- 11 years	0.96	0.95	0.97
	12+ years	0.76	0.75	0.77
Year of birth	2012	0.94	0.94	0.95
	2013	0.85	0.85	0.86
	2014	0.83	0.83	0.84
	2015	0.83	0.82	0.83
Age of mother		1.00	1.00	1.00
Relationship status	Single[§]	1.06	1.05	1.06
Ethnicity	Asian	0.98	0.97	0.99
	Black	1.07	1.05	1.08
	Indigenous	0.99	0.96	1.02
	Mixed	1.03	1.02	1.04
Previous Event		1.68	1.68	1.68

[§] Single refers to those who are not in a relationship, separated, divorced or widowed.

© 2025 Paixão ES et al. *JAMA NetworkOpen*.

eTable 21. Adjusted Cox proportional hazards model comparing Syphilis exposure for time to readmission from a complete-case analysis with low (<2500g) and high (≥6,000g) birth weight excluded (N= 9,604,227 records).

**Exposure status**		**Hazard ratio**	**95% Confidence Interval**	
Exposure				
Congenital vs Not Exposed		2.05	2.03	2.07
Maternal vs Not Exposed		1.40	1.38	1.41
Congenital or Maternal vs Not Exposed		2.05	2.03	2.07
Adjusted variables				
Region	North-East	0.97	0.97	0.98
	North	0.96	0.95	0.96
	South-East	1.02	1.01	1.02
	South	1.11	1.11	1.12
Mother's Education	1-3 years	1.00	0.98	1.01
	4-7 years	1.02	1.01	1.03
	8- 11 years	0.97	0.96	0.99
	12+ years	0.78	0.77	0.79
Year of birth	2012	0.95	0.95	0.95
	2013	0.86	0.85	0.86
	2014	0.84	0.84	0.84
	2015	0.83	0.83	0.83
Age of mother		1.00	1.00	1.00
Relationship status	Single[§]	1.05	1.05	1.06
Ethnicity	Asian	0.98	0.97	0.99
	Black	1.06	1.04	1.07
	Indigenous	0.98	0.96	1.01
	Mixed	1.02	1.01	1.03
Previous Event		1.68	1.68	1.68

[§] Single refers to those who are not in a relationship, separated, divorced or widowed.

© 2025 Paixão ES et al. *JAMA NetworkOpen*.

3.4) Time to death

eTable 22. Adjusted Cox proportional hazards model comparing Syphilis exposure for time to death from a complete-case analysis with preterm births excluded (N= 6,197,689 patients).

**Exposure status**		**Hazard ratio**	**95% Confidence Interval**	
Exposure				
Congenital vs Not Exposed		1.82	1.62	2.04
Maternal vs Not Exposed		0.91	0.76	1.09
Congenital or Maternal vs Not Exposed		1.29	1.15	1.43
Adjusted variables				
Region	North-East	1.10	1.07	1.13
	North	1.23	1.19	1.28
	Central-West	1.08	1.03	1.13
	South	0.97	0.93	1.01
Mother's Education	1-3 years	0.71	0.66	0.77
	4-7 years	0.55	0.51	0.59
	8- 11 years	0.39	0.36	0.42
	12+ years	0.30	0.27	0.33
Year of birth	2012	0.98	0.95	1.02
	2013	0.95	0.92	0.99
	2014	0.97	0.93	1.00
	2015	1.00	0.97	1.04
Age of mother		1.00	1.00	1.00
Relationship status	Single[§]	1.13	1.10	1.15
Ethnicity	Asian	0.39	0.36	0.42
	Black	0.52	0.48	0.57
	Indigenous	0.51	0.41	0.62
	Mixed	0.45	0.42	0.48

[§] Single refers to those who are not in a relationship, separated, divorced or widowed.

© 2025 Paixão ES et al. *JAMA NetworkOpen*.

eTable 23. Adjusted Cox proportional hazards model comparing Syphilis exposure for time to death from a complete-case analysis with low (<2500g) and high (≥6,000g) birth weight excluded (N= 6,709,819 patients).

**Exposure status**		**Hazard ratio**	**95% Confidence Interval**	
Exposure				
Congenital vs Not Exposed		1.81	1.62	2.04
Maternal vs Not Exposed		1.02	0.86	1.21
Congenital or Maternal vs Not Exposed		1.36	1.23	1.51
Adjusted variables				
Region	North-East	1.10	1.07	1.13
	North	1.24	1.20	1.29
	Central-West	1.10	1.05	1.14
	South	0.97	0.93	1.01
Mother's Education	1-3 years	0.71	0.66	0.76
	4-7 years	0.54	.050	0.58
	8- 11 years	0.38	0.36	0.41
	12+ years	0.29	0.26	0.31
Year of birth	2012	0.99	0.95	1.02
	2013	0.96	0.93	0.99
	2014	0.98	0.95	1.01
	2015	1.02	0.99	1.06
Age of mother		1.00	1.00	1.00
Relationship status	Single[§]	1.14	1.12	1.17
Ethnicity	Asian	0.39	0.36	0.42
	Black	0.52	0.48	0.56
	Indigenous	0.43	0.35	0.54
	Mixed	0.44	0.42	0.48

[§] Single refers to those who are not in a relationship, separated, divorced or widowed.

© 2025 Paixão ES et al. *JAMA NetworkOpen*.

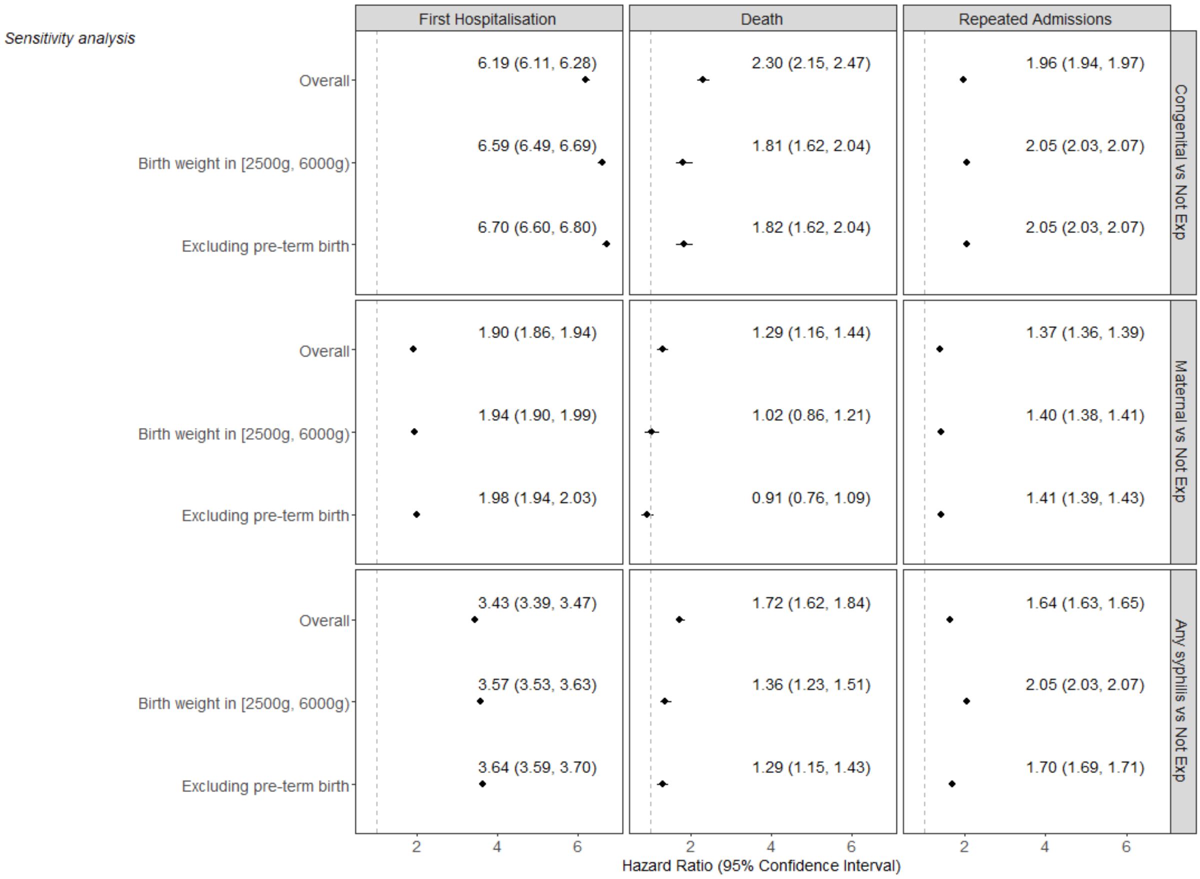

eFigure 4. Forest plot showing adjusted hazard ratios for (i) the main analysis, (ii) when excluding children with a birth weight under 2,500g or over 6,000g, and (iii) excluding preterm birth (<37 weeks)

© 2025 Paixão ES et al. *JAMA NetworkOpen*.

4) eReferences

1. Paixao ES, Cardim LL, Falcao IR, et al. Cohort Profile: Centro de Integração de Dados e Conhecimentos para Saúde (CIDACS) Birth Cohort. *Int J Epidemiol*. 2021;50(1):37-38. doi:10.1093/ije/dyaa255

2. Cerquiera DRC, Alves PP, Coelho DSC, Reis MVM, Lima AS. *Uma análise da base de dados do sistema de informação hospitalar entre 2001 e 2018: dicionário dinâmico, disponibilidade dos dados e aspectos metodológicos para a produção de indicadores sobre violência*. Ipea; 2019.

3. Ministério da Saúde. SIM. Sistema de Informações sobre Mortalidade. gov.br. Accessed September 4, 2024. https://svs.aids.gov.br/daent/cgiae/sim/documentacao/

© 2025 Paixão ES et al. *JAMA NetworkOpen*.
