# Supplementary material for: Syphilis Exposure During Pregnancy and Childhood Hospital Admissions in Brazil
Source: JAMA Netw Open. 2025 Apr 30;8(4):e257471. doi: 10.1001/jamanetworkopen.2025.7471 (PMC12044516; doi:10.1001/jamanetworkopen.2025.7471)
Supplement: Supplement 2. — Data Sharing Statement [file jamanetwopen-e257471-s002.pdf]

Data Sharing Statement

Paixão. Syphilis Exposure During Pregnancy and Childhood Hospital Admissions in Brazil. *JAMA Netw Open*. Published April 30, 2025. doi:10.1001/jamanetworkopen.2025.7471

Data
Data available: No

Additional Information
Explanation for why data not available: All data supporting the findings presented here were obtained from Centro de Integração de Dados e Conhecimentos para Saúde (CIDACS). Importantly, restrictions apply to the availability of these data. However, upon reasonable request and provided all ethical and legal requirements are met, the institutional data curation team can make the data available. Information on how to apply to access the data can be found at https://cidacs.bahia.fiocruz.br/en/.
